# Evaluation of the Elemental Composition, Identification and Quantification of Major Phenolic Compounds and Fatty Acids in Perennial *Salicornia* Species

**DOI:** 10.3390/foods15213774

**Published:** 2026-10-22

**Authors:** Vicenta de la Fuente, Lourdes Rufo, Esteban Ramírez, Nuria Rodríguez, Carlos Pernas, Ricardo Amils

**Affiliations:** 1 Universidad Autónoma de Madrid (UAM), Campus Cantoblanco, 28049 Madrid, Spain; carlos.pernas@uam.es; 2 Instituto de Investigaciones Biosanitarias, Facultad de Ciencias Experimentales, Universidad Francisco de Vitoria (UFV), Pozuelo de Alarcón, 28223 Madrid, Spain; 3 Botany Unit, Department of Pharmacology, Pharmacognosy and Botany, Faculty of Pharmacy, Complutense University of Madrid, Plaza de Ramón y Cajal s/n, 28040 Madrid, Spain; esrami02@ucm.es or esteban_ramirezchueca@fas.harvard.edu; 4 Department of Organismic and Evolutionary Biology, Harvard University Herbaria, Cambridge, MA 02138, USA; 5 Centro de Astrobiología (INTA-CSIC), Torrejón de Ardoz, 28850 Madrid, Spain; nrodriguez@cbm.csic.es; 6 Centro de Biología Molecular Severo Ochoa (UAM-CSIC), Cantoblanco, 28049 Madrid, Spain; ramils@cbm.csic.es

**Keywords:** elemental composition, fatty acids, phenols, seeds, *Salicornia*

## Abstract

Perennial *Salicornia* species are promising halophytic food resources, but comparative information on their mineral and bioactive composition remains limited. We characterized elemental composition, total phenolic content, individual phenolic compounds in aerial tissues and seeds, fatty acid profiles, and seed morphology in selected taxa from seven perennial *Salicornia* species collected across Mediterranean, Atlantic, and inland saline habitats. Elemental composition was assessed by ICP-MS in 68 samples from 18 sites. Na, K, Mg, and Ca dominated the elemental profile, while species-related differences were detected for K, Ca, Fe, Ni, and Ba; fruiting samples showed lower Fe, As, Sr, Ba, and Pb concentrations. Total phenolic content ranged from 2.579 to 3.924 mg GAE/g DW. Several glycosylated flavonoids were identified: isoquercetin and rutin were the major forms, whereas luteolin-7-*O*-glucoside and apigenin-7-*O*-glucoside occurred only sporadically. In seeds, rutin and isoquercetin together accounted for 65.1–96.2% of quantified phenolics. Eleven fatty acids were identified, with substantial contributions of linoleic and α-linolenic acids in several samples. These results expand the compositional and taxonomic knowledge of perennial *Salicornia* and support their potential as alternative food resources.

## 1. Introduction

The genus *Salicornia* L. (Salicornioideae Ulbr., Amaranthaceae Juss.) currently comprises 56 accepted taxa according to the taxonomic treatment adopted by the Plants of the World Online database [1]. Although some authors continue to recognize *Salicornia* and *Sarcocornia* as separate genera [2,3,4], the present study follows the POWO classification, in which both the annual species (*Salicornia*) and the perennial species (*Sarcocornia*) are included within the genus *Salicornia* [5]. These taxa are halophytes, plants adapted to environments with high salt concentrations, such as coastal salt marshes and inland saline lakes. Species identification in the field is particularly challenging because of their high morphological similarity, which has resulted in frequent taxonomic confusion and inconsistent treatments among national floras. This taxonomic uncertainty also has practical implications beyond field studies, particularly in the context of the increasing international trade in seeds. Accurate identification is therefore essential for determining the origin and taxonomic identity of commercially traded plant material.

Species of *Salicornia* and *Sarcocornia* have long been consumed by coastal communities and, more recently, have gained considerable attention as premium gastronomic ingredients. Because of their close morphological resemblance, both groups are frequently marketed and consumed under the same commercial name. Annual *Salicornia* species are currently the most commonly consumed and are widely marketed as marsh samphire or sea asparagus because of their valued flavor and texture [6]. However, perennial species may also be of considerable interest because their higher productivity and year-round availability could facilitate their cultivation and commercialization. Accordingly, a better understanding of their nutritional and bioactive profiles is essential for assessing their potential in sustainable food production [7].

Ethnobotanical studies conducted throughout Europe, North Africa, and several Asian regions have emphasized the importance of *Salicornia* species in traditional diets and folk medicine, reflecting a rich body of local ecological knowledge [4,8]. These halophytes are recognized as valuable sources of nutrients, particularly minerals and dietary fiber, as well as a wide range of bioactive compounds, including fatty acids, and phenolic compounds, like flavonoids, which are largely responsible for their health-promoting properties [9,10,11]. Evidence from both in vitro experiments and animal models indicates that their extracts may contribute to glycemic control by improving insulin sensitivity, highlighting their potential as natural alternatives or complementary agents to conventional antidiabetic therapies [12,13]. Although *Salicornia europaea* remains the most extensively investigated species [14], increasing attention has recently been directed towards the perennial ones, such as *Salicornia fruticosa* [15] and *Salicornia perennis* [8,10], particularly regarding their antioxidant and antidiabetic properties and their potential as sources of health-promoting phytochemicals.

Recent studies by our research team have shown that *Salicornia* and related genera can accumulate substantial concentrations of essential macro- and micronutrients, including Na, K, Mg, Ca, Fe, Mn, and Zn [16,17,18]. The association of several of these elements with distinct crystalline mineral phases suggests that biomineralization contributes to nutrient sequestration and ion homeostasis under saline conditions. Fe is particularly noteworthy, as its accumulation has been associated with crystalline iron phases and electron-dense structures consistent with ferritin iron cores in chloroplasts [17,18]. Overall, the coexistence of crystalline mineral phases and chloroplast-localized ferritin supports the role of complementary mineral-and protein-based mechanisms in maintaining nutrient homeostasis and protecting against ionic and oxidative stress in these halophytes.

In the present study, we investigated seven perennial *Salicornia* species that are frequently misidentified, namely *S. alpini*, *S. carinata*, *S. hispanica*, *S. fruticosa*, *S. lagascae*, *S. perennis*, and *S. pruinosa*. Plant material was collected from several locations across the western Mediterranean region and adjacent Atlantic coasts, including Spain, Portugal, France, Sardinia (Italy), and Tunisia. The main objective of this study was to characterize the macro- and micronutrient composition of the profile of bioactive compounds including fatty acids, and phenolic compounds in these species, while extending the phenolic characterization to seeds. This approach allowed us to evaluate their nutritional value and their potential as emerging food resources for human consumption.

## 2. Materials and Methods

### 2.1. Sample Collection and Preparation

Specimens of *Salicornia alpini*, *S. carinata*, *S. hispanica*, *S. fruticosa*, *S. lagascae*, *S. perennis*, and *S. pruinosa* were collected from the populations listed in Table 1.

Sampling was conducted at different times of the year over several consecutive years. Voucher specimens were prepared for all collections and deposited in the Faculty of Pharmacy of the Complutense University of Madrid Herbarium (MAF). Most collections were made during the fruiting season (autumn and winter) between 2019 and 2025, but vegetative and flowering samples were also collected.

Analyzed plant material was vegetative (succulent photosynthetic stems) with or without flowers and fruits. For seed analyses, seeds were carefully separated from the infructescence, cleaned by sieving, and manually sorted to remove damaged seeds and other impurities.

Samples intended for these chemical analyses were processed as follows. Fresh samples were dried in a forced-air oven at 40 ± 5 °C for 35 h, ground to a particle size of ≤1 mm, thoroughly homogenized, and stored in sterile, airtight plastic containers at 4 °C until chemical analysis.

### 2.2. Seed Optical Stereomicroscopy

Seed morphological and micromorphological characterization was performed by optical stereomicroscopy. The Olympus SZX10 stereomicroscope, fitted with an Olympus SC30 camera (Olympus Corporation, Tokyo, Japan), was used to take photographs. Measurements were made using ImageJ (Java 1.8.0).

### 2.3. Inductively Coupled Plasma Mass Spectrometry (ICP-MS)

These analyses were carried out using the optimized methodology reported by [19]. Approximately 500 mg of dry powered plant material was digested in a Teflon tube in a mixture of 8 mL of 65% HNO_3_ and 2 mL of 30% H_2_O_2_ inside an MLS Ethos 1600 URM Milestone high-pressure microwave digester (Milestone S.r.l., Sorisole, Italy). The volume of each sample was subsequently adjusted to 25 mL with deionized water.

Na, Mg, K, Ca, Mn, Fe, Ni, Cu, Zn, As, Sr, Ba and Pb concentrations were determined in aliquots of the solutions obtained for each sample by ICP-MS using an ICP ELAN-600 PE Sciex Instrument (Toronto, ON, Canada) and expressed as mg kg^−1^ dry weight (DW). This technique has an inherent error of 15%.

### 2.4. Total Phenolic Compounds (TPCs)

Total phenolic content was determined by the Folin–Ciocalteu method [20] using gallic acid as the recommended standard [21]. A 0.5 mL aliquot of methanolic extract was taken from the extracts obtained previously, and 2.5 mL of Folin–Ciocalteu reagent was added, and left to react for 3 min. Then, 2 mL of Na_2_CO_3_ solution was added and mixed in a Heidolph shaker (Berlin, Germany). The solution was incubated at a temperature of 40 °C and stored in the dark for 1h. The absorbance was measured at 765 nm with a spectrophotometer, and the results were expressed as gallic acid equivalents (G.A.E.s).

### 2.5. Determination of Bioactive Compounds

#### 2.5.1. Preparation of Methanol Extract

Five hundred milligrams of dried plant sample were extracted with a solution of 40 mL of methanol at 25 °C. It was kept in magnetic stirring for 60 min. The extracts were filtered using a Whatman No. 4 filter. The solid residue was recovered and extracted with 40 mL methanol. The extracts were filtered again and combined and evaporated (35 °C under vacuum of the methanolic extracts), then redissolved with methanol to obtain a 30 mg/mL extract solution, from which different dilutions were made (from 0.03125 mg/mL to 16 mg/mL). The extractions were performed in triplicate and were stored at 4 °C until the execution of the analyses.

#### 2.5.2. Gas Chromatography Coupled with Mass Spectrometry (GC-MS) for Fatty Acid Analysis

Fatty acid analysis was performed using a 436GC gas chromatograph (Bruker Daltonics, Bremen, Germany) coupled to an EVOQ-TQ Premium EI&CI mass spectrometer (Bruker Daltonics). Data acquisition and processing were carried out using Bruker Mass Spectrometry Workstation software (version 8.2.1).

Chromatographic separations were achieved on an Rxi-5Sil MS capillary column (30 m × 0.25 mm i.d., 0.25 μm film thickness; Restek, Bellefonte, PA, USA). Helium was used as the carrier gas at a constant flow rate of 1.0 mL min^−1^. Samples were injected using a COMBI-PAL autosampler (Bruker Daltonics GmbH & Co. KG, Bremen, Germany; serial number 328847) with an injection volume of 1.0 μL in split mode (1:100). The injector temperature was maintained at 280 °C.

The oven temperature program was as follows: initial temperature of 80 °C, held for 5 min; ramped to 150 °C at 20 °C min^−1^ and held for 20 min; then increased to 200 °C at 1 °C min^−1^ without a holding step; finally increased to 300 °C at 10 °C min^−1^ and held for 10 min. The total run time was 98.5 min.

Mass spectrometric detection was performed using electron ionization (EI) at 70 eV. Mass spectra were acquired over an *m*/*z* range of 40–650. The transfer line and ion source temperatures were maintained at 250 °C and 200 °C, respectively. A solvent delay between 5 and 15 min was applied depending on the analytical sequence.

Fatty acid identification was accomplished by comparison of retention times and mass spectra with those obtained from the Supelco 37 Component FAME Mix standard (Sigma-Aldrich, St. Louis, MO, USA). Additional confirmation was achieved through spectral matching with the NIST mass spectral library. Quantification was carried out using external standard calibration curves for each identified compound. Calibration standards were prepared from authentic commercial reference materials, and analyte concentrations in the samples were calculated from the corresponding calibration curve.

The limits of detection (LOD) and quantification (LOQ) were not experimentally determined in the present study. Quantification was performed using external standard calibration curves for each analyte. Since the limits of detection (LOD) and quantification (LOQ) were not experimentally established, the lowest calibration point of the external standard calibration curve was considered as the practical lower limit of quantification (LOQ) for each analyte. This level corresponded to 50 ng for most fatty acid methyl esters (FAMEs), whereas for C24:0 and C18:3, it was 250 ng.

#### 2.5.3. High-Performance Liquid Chromatography–Electrospray Ionization Mass Spectrometry (HPLC-MS/ESI) for Flavonoid and Hydroxycinnamic Acids Analysis

Flavonoids were analyzed by high-performance liquid chromatography coupled to tandem mass spectrometry (HPLC-MS/MS) using an EVOQ Elite mass spectrometer (Bruker, Bremen, Germany) equipped with an electrospray ionization (ESI) source. Chromatographic separation was performed on an ACE 3 C18 column (150 × 3.0 mm, 3 μm particle size) maintained at 25 °C.

The mobile phase consisted of water containing 0.1% acetic acid (solvent A) and acetonitrile containing 0.1% acetic acid (solvent B). The gradient elution program was as follows: 0.0 min, 90% A; 0.5 min, 90% A; 5.0 min, 50% A; 10.0 min, 50% A; 15.0 min, 0% A; 17.0 min, 0% A; 17.2 min, 90% A; and 20.0 min, 90% A. The flow rate was 0.4 mL min^−1^.

Mass spectrometric detection was carried out in negative electrospray ionization mode (ESI−) using a targeted MS/MS method. Compound identification was based on the characteristic fragmentation patterns obtained from the corresponding deprotonated molecular ions ([M−H]^−^). The ion source parameters were set as follows: source type, HESI; spray voltage, −4500 V (negative mode); cone temperature, 350 °C; cone gas flow, 40 arbitrary units; heated probe temperature, 400 °C; probe gas flow, 50 arbitrary units; nebulizer gas flow, 60 arbitrary units; and exhaust gas, ON.

Phenolic compounds, flavonoids, and hydroxycinnamic acids were analyzed by HPLC-ESI-MS/MS using a triple quadrupole mass spectrometer operating in multiple reaction monitoring (MRM) mode. Compound identification was achieved by comparison with authentic commercial standards. For each analyte, the precursor-to-product ion transitions, collision energies (CEs), and retention times under the chromatographic gradient conditions employed are summarized in Appendix A.

Since the mass spectrometer used was a unit-resolution triple quadrupole instrument, identification was based on the combination of retention time and characteristic MRM transitions. As analyses were performed using a low-resolution mass spectrometer, mass error criteria in parts per million (ppm) were not used, since these parameters are typically reserved for high-resolution mass spectrometry measurements.

#### 2.5.4. Calculation of Fatty Acid Nutritional Quality Indices

Nutritional quality indices were calculated from the relative abundance of individual fatty acids. The PUFA/SFA ratio was calculated as the sum of polyunsaturated fatty acids divided by the sum of saturated fatty acids and provides a general measure of the relative contribution of these two fatty-acid classes. The index of atherogenicity (IA) was calculated as [C12:0 + 4(C14:0) + C16:0]/[ΣMUFA + ΣPUFA], giving greater weight to the saturated fatty acids considered to have the highest atherogenic potential. The hypocholesterolemic/hypercholesterolemic ratio (h/H) was calculated as [C18:1 + ΣPUFA]/[C12:0 + C14:0 + C16:0] and expresses the balance between fatty acids generally considered favorable and those associated with hypercholesterolemic effects. Higher PUFA/SFA and h/H values and lower IA values indicate a more favorable fatty-acid profile.

### 2.6. Descriptive and Graphical Analyses

#### 2.6.1. ICP Data

The dataset comprised 68 analytical portions from 46 independent plants representing seven *Salicornia* species collected at 18 sampling sites. The individual plant was considered the statistical unit. When multiple portions were available, their valid elemental concentrations were averaged arithmetically before statistical analysis. Concentrations were expressed as mg kg^−1^ dry weight (DW). Instrumental non-detects (reported as 0.000) were treated as missing values and were not imputed. Consequently, 46 plants were available for 11 elements, 45 for Pb, and 30 for As.

Arithmetic means, standard deviations (SDs), medians, and ranges were calculated overall and by species. Absolute elemental profiles were represented by species medians and interquartile ranges (Q1–Q3) on a logarithmic scale. Relative profiles were calculated as log_2_(species median/genus reference), where the genus reference was the geometric mean of the seven species-specific medians for each element, thereby assigning equal weight to each species.

#### 2.6.2. Statistical Analysis for ICP-Data

Element-specific concentrations were analyzed after log_10_ transformation. Interspecific differences were assessed using linear mixed-effects models (LMMs), with species as a fixed effect and sampling site as a random intercept. Models were fitted by restricted maximum likelihood (REML). For As, a linear model (LM) was used because the estimated random-intercept variance was zero. Global species effects were evaluated using F tests, with Satterthwaite denominator degrees of freedom for LMMs. The resulting 13 *p* values were adjusted using the Benjamini–Hochberg false discovery rate (FDR) procedure. Model-estimated marginal means and 95% confidence intervals (CIs) were back-transformed to obtain geometric mean concentration estimates. Pairwise comparisons were considered exploratory rather than confirmatory.

Phenological associations were assessed using additive models including species and phenological stage (vegetative or fruiting) as fixed effects and sampling site as a random intercept, except for As, which was analyzed using an LM adjusted for species. Species × phenology interactions were not included because of incomplete representation of phenological stages across species. Effects were expressed as fruiting-to-vegetative concentration ratios with 95% CIs. A separate Benjamini–Hochberg correction was applied to the 13 phenological tests. Statistical significance was defined as FDR-adjusted *p* < 0.05; confidence intervals were not multiplicity-adjusted.

Model convergence, singularity, and residual distributions were examined using numerical diagnostics, residual-versus-fitted plots, and Q–Q plots. Potentially influential observations and sampling sites were assessed through targeted sensitivity analyses.

For the phenological association with As, leave-one-plant-out analysis was performed by refitting the species-adjusted LM after excluding each of the 30 plants individually. An additional analysis was restricted to 11 plants from four species–site combinations containing both phenological stages, using an LM with species–site combination and phenology as fixed effects.

Statistical analyses were performed using R version 4.6.1 (R Core Team, 2026) within the RStudio integrated development environment (version 2026.07.1, Build 147; Posit Software, PBC). Linear mixed-effects models were fitted using the lme4 (version 2.0-6) and lmerTest (version 3.2-1) packages, and estimated marginal means were calculated using emmeans (version 2.0.4). Data manipulation and visualization were performed using dplyr (version 1.2.1), tidyr (version 1.3.2), and ggplot2 (version 4.0.3).

#### 2.6.3. Phenolic Compounds Data

For each sample of plant tissues, two technical replicates were averaged. For *Salicornia fruticosa* (SFRU_NAB_02), chlorogenic acid, hydroxytyrosol, and salicylic acid concentrations were based on a single valid measurement because of an injection failure in the second replicate.

Total quantified phenolic content was calculated as the sum of the mean concentrations of the 18 compounds analyzed and expressed in ng/mg. The relative abundance of each compound was calculated as a percentage of the total quantified content. Given its predominance across samples, isoquercetin was also compared with the combined concentration of the remaining 17 compounds.

For each sample of seeds, two technical replicates were averaged. The total quantified content was calculated as the sum of the concentrations of quercetin, rutin, isoquercetin, and salicylic, ferulic, and chlorogenic acids, expressed in ng/mg. The relative contribution of each compound was expressed as a percentage of that sum, and the combined contribution of rutin and isoquercetin was calculated using the unrounded values. Differences in total content were expressed as the ratio of the highest to the lowest value.

## 3. Results

### 3.1. Elemental Composition and ICP-MS Analysis

#### 3.1.1. Dataset and Overall Elemental Composition

The dataset comprised 68 analytical portions from 46 independent plants representing seven perennial *Salicornia* species collected at 18 sampling sites (Table 2). The number of plants varied among species, ranging from two in *S. perennis*, *S. lagascae*, and *S. fruticosa* to 24 in *S. carinata*. Twenty-seven plants were classified as vegetative and 19 as fruiting. Both phenological stages were represented in five species, whereas *S. perennis* was represented exclusively by vegetative plants and *S. lagascae* by fruiting plants.

**Figure 1:**
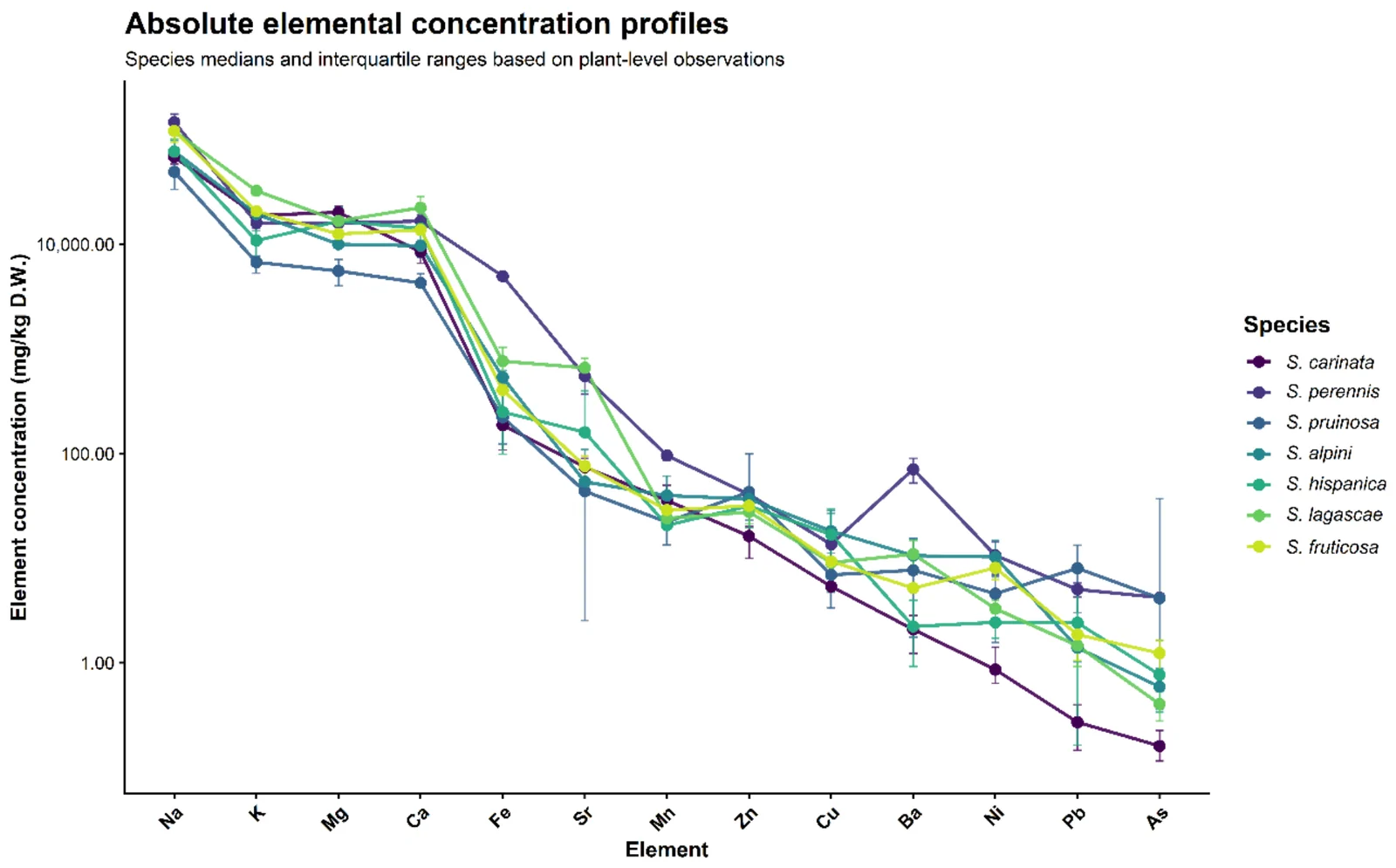
Absolute elemental concentration profiles of the seven *Salicornia* species. Points represent species-specific median concentrations, and error bars indicate the interquartile range (Q1–Q3), calculated from plant-level observations. When multiple analytical portions were available for a plant, concentrations were first averaged on the original scale. Elements are ordered from highest to lowest overall median concentration in the complete genus dataset. Element concentrations are expressed as mg kg^−1^ dry weight (DW) and plotted on a logarithmic scale. Lines connect medians for visual guidance only.

The overall elemental composition was dominated by Na, K, Mg, and Ca (Table 3; Figure 1). Median concentrations were 72,132 mg kg^−1^ DW for Na, 17,014 for K, 16,560 for Mg, and 8536 for Ca. The remaining elements decreased in plant tissues in the following order: Fe, Sr, Mn, Zn, Cu, Ba, Ni, Pb, and As.

Substantial variability was observed, particularly for As, Pb, and Fe, whose arithmetic means considerably exceeded their medians. The greatest discrepancies occurred for As (mean: 10.0; median: 0.425 mg kg^−1^ DW) and Pb (mean: 3.21; median: 0.553 mg kg^−1^ DW), reflecting strongly right-skewed distributions and the presence of high individual concentrations.

**Figure 2:**
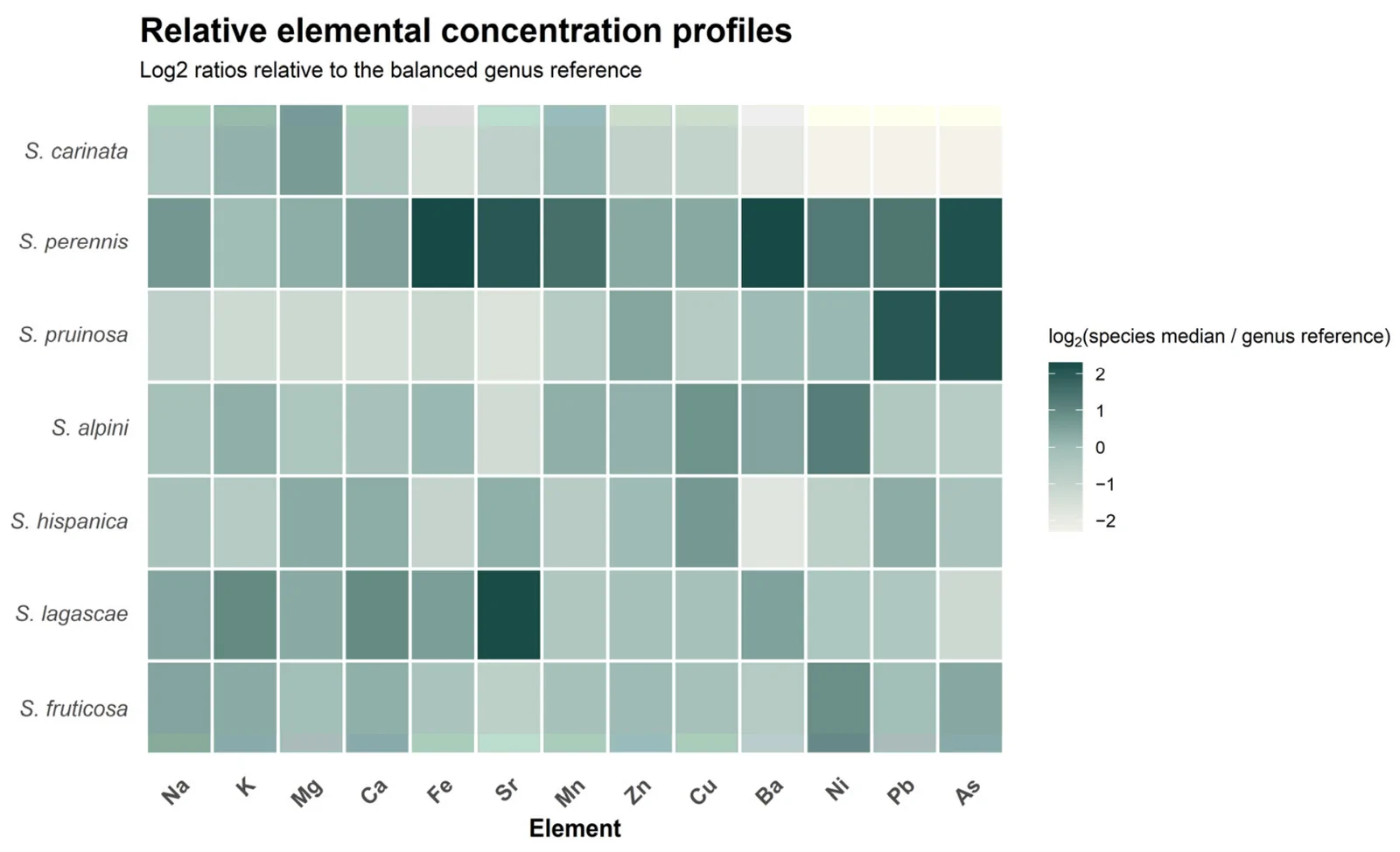
Relative elemental concentration profiles of the seven *Salicornia* species. Heatmap of log_2_-transformed ratios relative to a species-balanced genus reference, calculated as the median of the seven species-specific median concentrations. A value of 0 indicates that the species median equals the reference; positive values indicate relative enrichment (+1, two-fold; +2, four-fold), whereas negative values indicate relative depletion (−1, one-half; −2, one-quarter of the reference concentration).

Although Na was the predominant element in all seven species, their elemental concentration profiles showed considerable descriptive variation (Table 4; Figure 1 and Figure 2). *Salicornia perennis* exhibited particularly high median Fe and Ba concentrations, whereas *S. lagascae* showed the highest median K, Ca, and Sr concentrations. *Salicornia pruinosa* had the lowest median concentrations of all four major elements (Na, K, Mg, and Ca), together with the highest median Pb concentration. The species-balanced relative profiles (Figure 2) further highlighted elevated relative As and Pb concentrations in *S. pruinosa*, Fe and Ba in *S. perennis*, and Ni and Cu in *S. alpini*. These patterns describe the observed elemental profiles without implying statistically confirmed differences among species.

#### 3.1.2. Differences in Elemental Concentrations Among Species

Global tests indicated nominal species effects (*p* < 0.05) for K (F_6,12_._45_ = 3.85, *p* = 0.021), Ca (F_6,4_._98_ = 6.90, *p* = 0.026), As (F_6,23_ = 3.34, *p* = 0.016), and Ba (F_6,7_._33_ = 7.25, *p* = 0.009). However, none of these effects remained statistically significant after Benjamini–Hochberg correction across the 13 elements (FDR-adjusted *p* = 0.084 for all four). No nominal species effect was detected for the remaining nine elements (Table 5).

**Figure 3:**
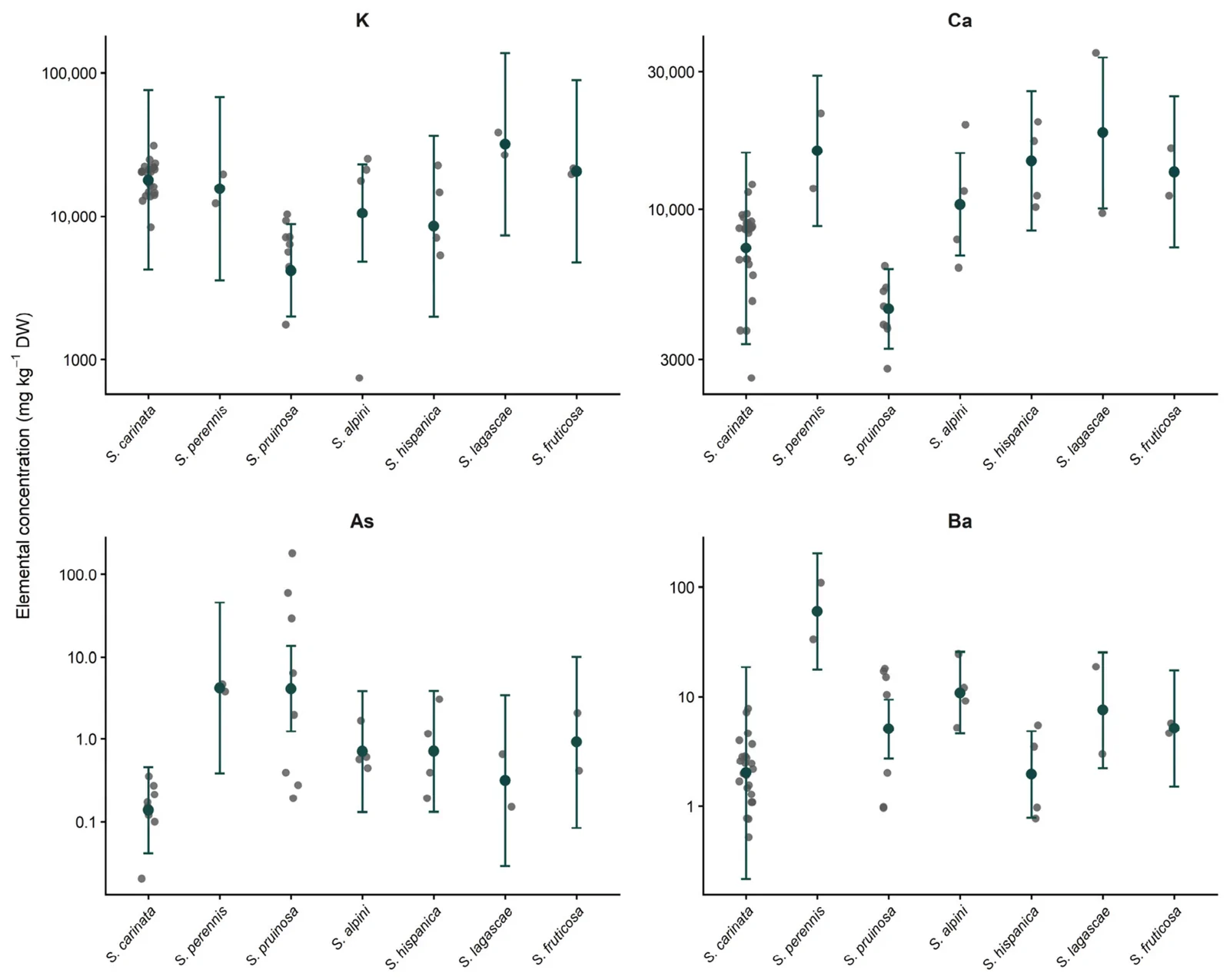
Plant-level concentrations and model-estimated marginal means for K, Ca, As, and Ba among the seven studied *Salicornia* species. Gray points represent individual plant-level observations. Dark green points and vertical bars represent model-estimated marginal means and their 95% confidence intervals, respectively. Elemental concentrations were analyzed after log10 transformation. Models included species as a fixed effect and sampling site as a random intercept, except for As, which was analyzed using a linear model because the estimated sampling-site variance was zero. Model estimates and confidence intervals were back-transformed to the original concentration scale. Concentrations are expressed as mg kg^−1^ dry weight (DW), and the *y*-axis is displayed on a logarithmic scale.

Model-estimated marginal means nevertheless revealed substantial variation among species for K, Ca, As, and Ba (Figure 3; Appendix B). Estimated K concentrations ranged from 4175 mg kg^−1^ DW in *S. pruinosa* to 31,930 mg kg^−1^ DW in *S. lagascae*, while the corresponding Ca estimates ranged from 4497 to 18,401 mg kg^−1^ DW. For As, the lowest estimated concentration was observed in *S. carinata* (0.137 mg kg^−1^ DW), whereas *S. perennis* and *S. pruinosa* showed higher estimates (4.19 and 4.10 mg kg^−1^ DW, respectively). The highest estimated Ba concentration was observed in *S. perennis* (59.8 mg kg^−1^ DW).

Confidence intervals were frequently wide, particularly for species represented by few independent plants, indicating considerable uncertainty in the estimated concentrations. Accordingly, these patterns were interpreted descriptively, and no pairwise interspecific differences were considered statistically confirmed following FDR correction.

#### 3.1.3. Associations Between Phenological Stage and Elemental Concentrations

Phenological stage was associated with differences in the model-estimated concentrations of several elements (Table 6). Nominally significant associations (*p* < 0.05) were observed for As, Sr, Ba, and Pb, with lower estimated concentrations in fruiting than in vegetative plants. The fruiting-to-vegetative concentration ratios were 0.110 for As (95% CI: 0.032–0.377; *p* = 0.0012), 0.423 for Sr (95% CI: 0.204–0.876; *p* = 0.0221), 0.488 for Ba (95% CI: 0.286–0.832; *p* = 0.0101), and 0.409 for Pb (95% CI: 0.202–0.827; *p* = 0.0145). However, after Benjamini–Hochberg correction across the 13 elements, only As remained statistically significant (FDR-adjusted *p* = 0.0157). The adjusted *p* values for Sr, Ba, and Pb were 0.0718, 0.0629, and 0.0629, respectively. No significant phenological association was detected for the remaining nine elements.

The association between phenological stage and As concentration was further examined through sensitivity analyses. All 30 leave-one-plant-out models retained nominal statistical significance (*p* < 0.05), with fruiting-to-vegetative concentration ratios ranging from 0.063 to 0.153, indicating that the association was not driven by any single observation. However, when the analysis was restricted to 11 plants from four species–site combinations containing both phenological stages, the estimated ratio remained below one (0.136), but the association was no longer statistically significant (95% CI: 0.010–1.881; *p* = 0.112).

The interpretation of the As association is constrained by the uneven distribution of phenological stages across species and sampling sites, together with the structured occurrence of non-detects. In particular, all 16 missing As measurements corresponded to *S. carinata*, leaving only eight plants of this species with valid As concentrations. Although the estimated association was consistent in direction across sensitivity analyses, the restricted comparison showed considerable uncertainty, limiting the strength of conclusions regarding phenological differences in As accumulation.

#### 3.1.4. Variability in Elemental Concentrations

Substantial variability in elemental concentrations was observed among individual plants, particularly for several trace elements (Table 3 and Table 4). This was especially evident in *S. pruinosa*, which showed pronounced dispersion in As (mean ± SD: 34.9 ± 62.6 mg kg^−1^ DW; median: 4.14 mg kg^−1^ DW) and Pb (12.9 ± 16.7 mg kg^−1^ DW; median: 8.00 mg kg^−1^ DW). The highest As concentration (181 mg kg^−1^ DW) was recorded in plant PRU_06 from Carabelas, whereas the highest Pb concentration (51.9 mg kg^−1^ DW) occurred in plant PRU_08 from Isla Cristina. Additional *S. pruinosa* plants from different sampling sites also exhibited elevated As concentrations, indicating that high As values were not restricted to a single individual.

Considerable uncertainty was also evident in several model-based estimates, as reflected by wide confidence intervals, particularly in species represented by few independent plants (Figure 3; Appendix B). Sampling was unevenly distributed among species and localities, with most sites represented by a single species. These features limited the precision of interspecific estimates and complicated the separation of species-related variation from differences associated with sampling sites.

### 3.2. Total Phenolic Content (TPC)

The TPC of the perennial *Salicornia* samples ranged from 2.579 to 3.924 mg GAE/g DW across populations from Atlantic and Mediterranean coastal areas of Spain and Tunisia and inland areas of central Spain (Table 7). TPC values were relatively consistent across the analyzed samples, with the highest value recorded in *S. hispanica* from Villena (SHIS_ALI_04) and the lowest in *S. lagascae* from Santa Pola (SLAG_ALI_03).

### 3.3. Phenolic Compounds

#### Plant Tissues

**Figure 4:**
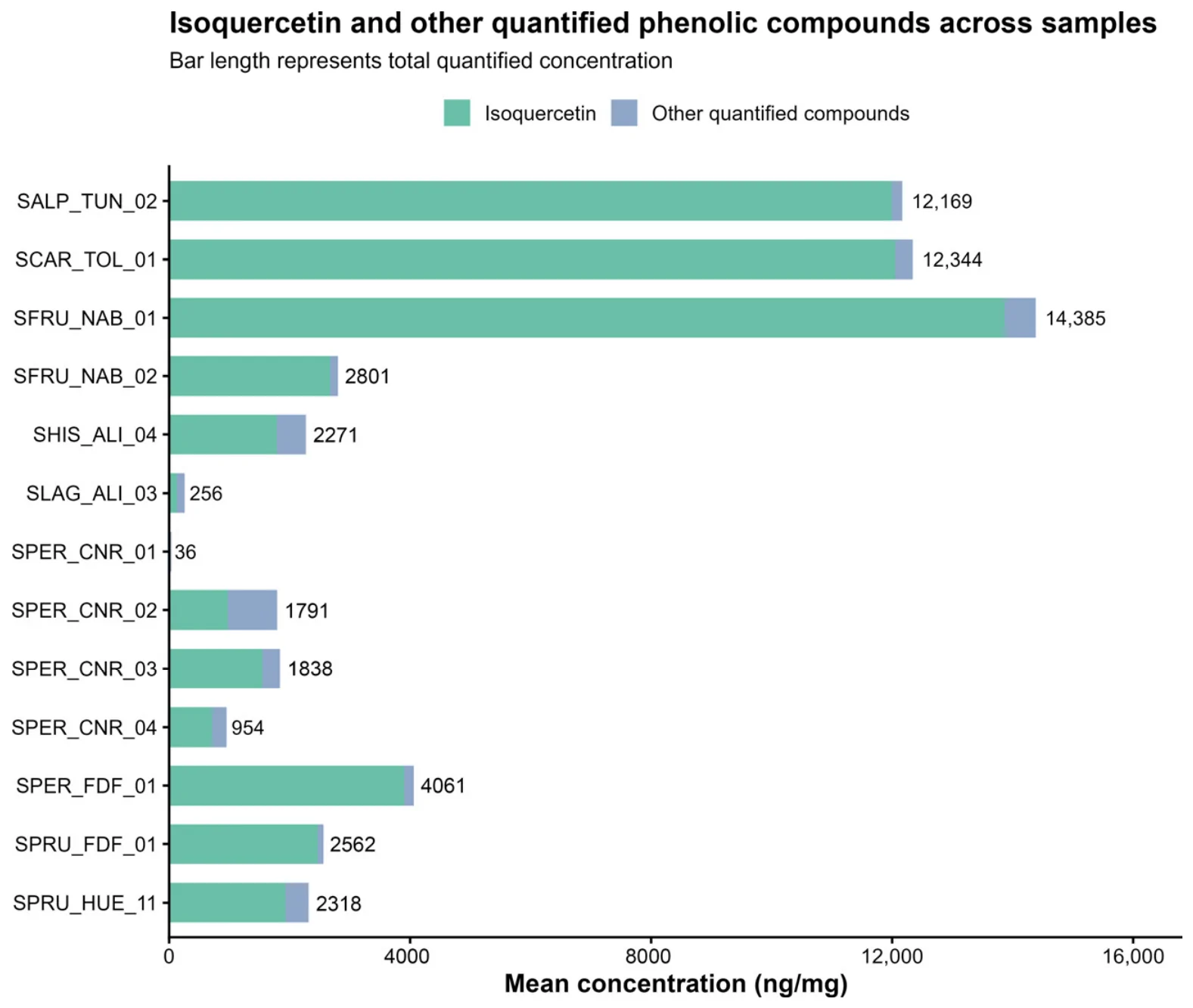
Concentration of isoquercetin and the combined concentration of the other 17 compounds in the samples analyzed. The total length of each bar represents the total quantified concentration.

**Figure 5:**
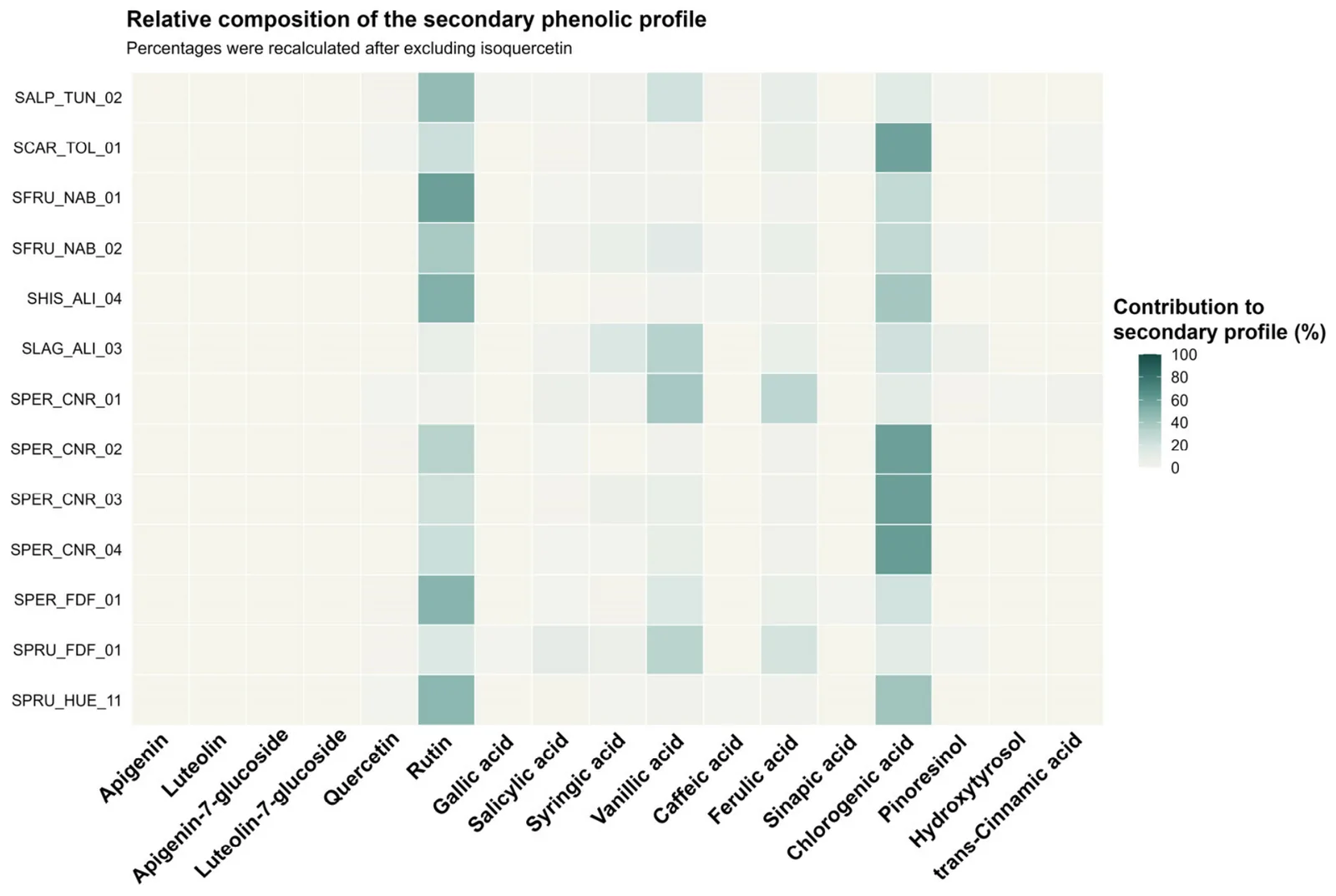
Relative composition of the phenolic profile after excluding isoquercetin. Percentages were calculated relative to the combined concentration of the remaining 17 compounds. The color scale represents the relative contribution (%) of each compound to this secondary profile.

Eighteen compounds were quantified in 13 samples representing seven species (Figure 4 and Figure 5). Total concentrations ranged from 36.1 ng/mg in *Salicornia perennis* from Corralejo (SPER_CNR_01) to 14,385.3 ng/mg in *S. fruticosa* from Korba (SFRU_NAB_01). Flavonoids were generally the predominant group, from 55.3% in *S. lagascae* (SLAG_ALI_03) to 99.3% in *S. alpini* (SALP_TUN_02). Conversely, phenolic acids were more variable and dominated only in SPER_CNR_01 (73.3% vs. 19.1%). Lignans and the remaining compounds made minor contributions to most samples.

Several glycosylated flavonoids were identified. Isoquercetin and rutin were widespread and quantitatively predominant, whereas luteolin-7-O-glucoside and apigenin-7-O-glucoside were detected only sporadically.

Isoquercetin was the predominant compound in 12 of the 13 samples, accounting for 51.6–98.6% of their total quantified content (Figure 4). The highest total concentrations were therefore largely attributed to this compound. The only exception was *S. perennis* from Corralejo (SPER_CNR_01), in which vanillic acid predominated and isoquercetin accounted for 19.1% of the total.

After isoquercetin, chlorogenic acid and rutin were the most abundant compounds, both occurring in all samples. The combined contribution of phenolic acids was particularly high in *S. lagascae* from Santa Pola (SLAG_ALI_03) and *S. perennis* from Órzola (SPER_CNR_02), accounting for 41.9% and 30.1% of the total, respectively. Excluding isoquercetin from the profile provided a clearer representation of the variation in less abundant compounds among samples (Figure 5).

### 3.4. Seeds

#### 3.4.1. Salicornia Seed Morphology

**Figure 6:**
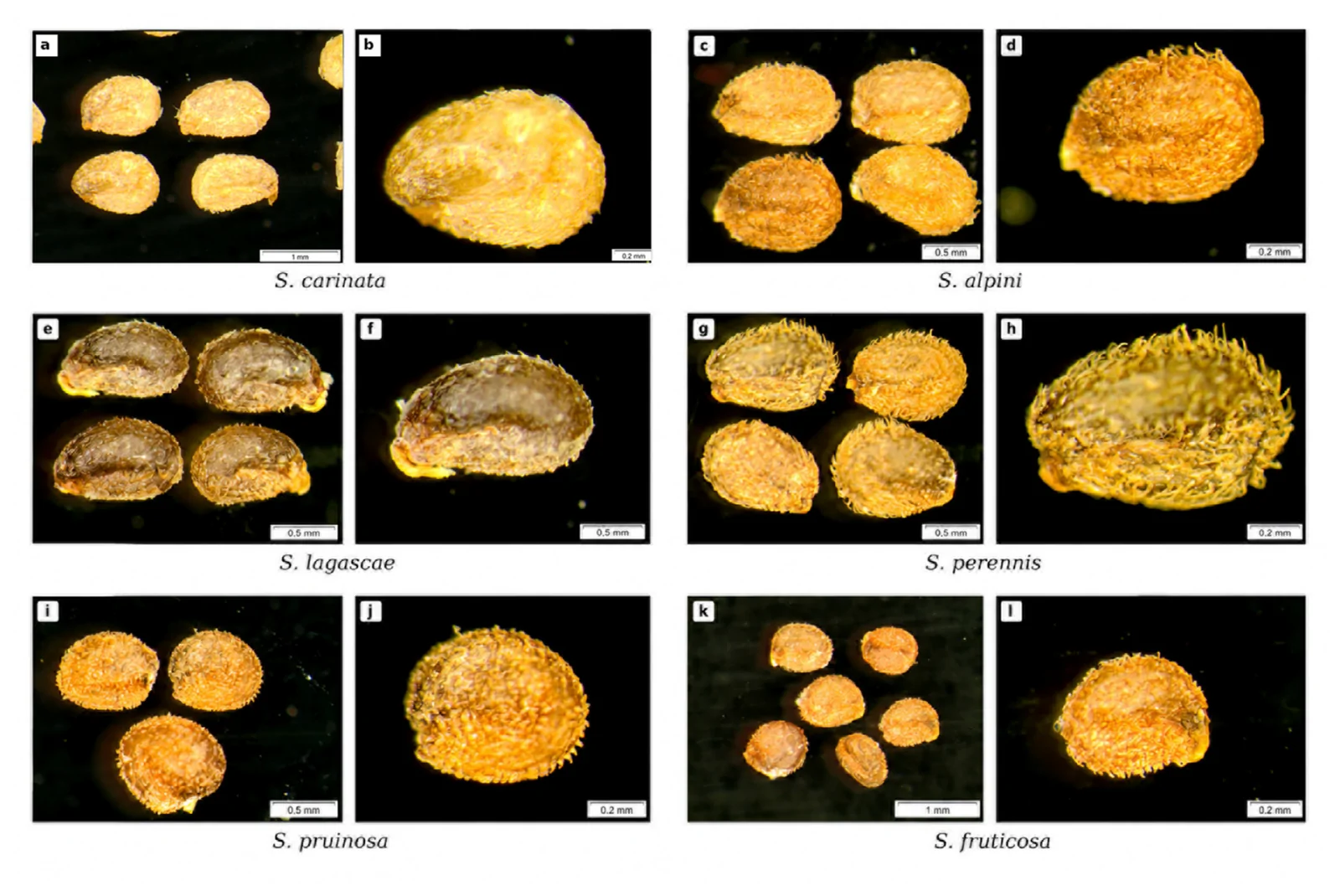
Optical images of seed detail of *S. carinata* (**a**,**b**) SCAR_TOL_11; *S. alpini* (**c**,**d**) SALP_TUN_01; *S. lagascae* (**e**,**f**) SLAG_MEN_01; *S. perennis* (**g**,**h**) SPER_HUE_01; *S. pruinosa* (**i**,**j**) SPRU_SAN_01; *S. fruticosa* SFRU_SAR_01 (**k**,**l**).

The genus *Salicornia* is widely distributed in salt marshes and saline inland habitats and includes annual and perennial species [22]. Seed testa ornamentation is a valuable micromorphological character for species discrimination [3]. As shown in Figure 6, *S. lagascae* has a verrucate testa with rounded and conical papillae, *S. fruticosa* has short hair mainly along the margins, and *S. pruinosa* has short hairs covering the entire seminal surface. *S. perennis* and *S. alpini* are characterized by long, dense hairs. Here, we recognize Salicornia at species rank, following the nomenclatural combination previously proposed in [23]. This taxon differs from *S. alpini* in its strongly carinated leaves and seed characters. *S. carinata* has significantly smaller seeds and shorter, thinner ap-pressed trichomes (mean length and width: 53.8 and 17.4 µm, respectively) than *S. alpini* (88.9 and 21.7 µm, respectively) [3].

#### 3.4.2. Phenolic Compounds in Seeds

Six phenolic compounds were quantified in the seeds: the flavonoids quercetin, rutin, and isoquercetin, and the phenolic acids salicylic, ferulic, and chlorogenic (Table 8). The profiles were dominated by rutin and isoquercetin, which together accounted for between 65.1% and 96.2% of the total of the six quantified compounds. The total content ranged from 1618 ng/mg in *S. pruinosa* from the Río Tinto estuary (SPRU_HUE_08) to 10,790 ng/mg in *S. fruticosa* from Sardinia (SFRU_SAR_01).

*S. alpini* sample (SALP_TUN_01) was dominated by rutin and isoquercetin, which accounted for 44.5% and 51.7% of the total quantified content, respectively, and together reached 96.2%. In contrast, the *S. carinata* samples exhibited contrasting profiles. At Laguna Larga (SCAR_TOL_10), isoquercetin predominated, accounting for 54.6%, while at Laguna de Tírez (SCAR_TOL_11), rutin predominated, accounting for 67.6%, accompanied by greater contributions from ferulic and chlorogenic acids.

The five *S. pruinosa* samples were dominated by rutin, whose contribution ranged from 58.5% to 64.3%. The main differences among populations were observed for isoquercetin, ranging from 6.0% to 29.6%, and for ferulic and chlorogenic acids. Overall, the samples differed both in their total content and in the relative composition of the six quantified compounds.

### 3.5. Fatty Acid Composition

**Figure 7:**
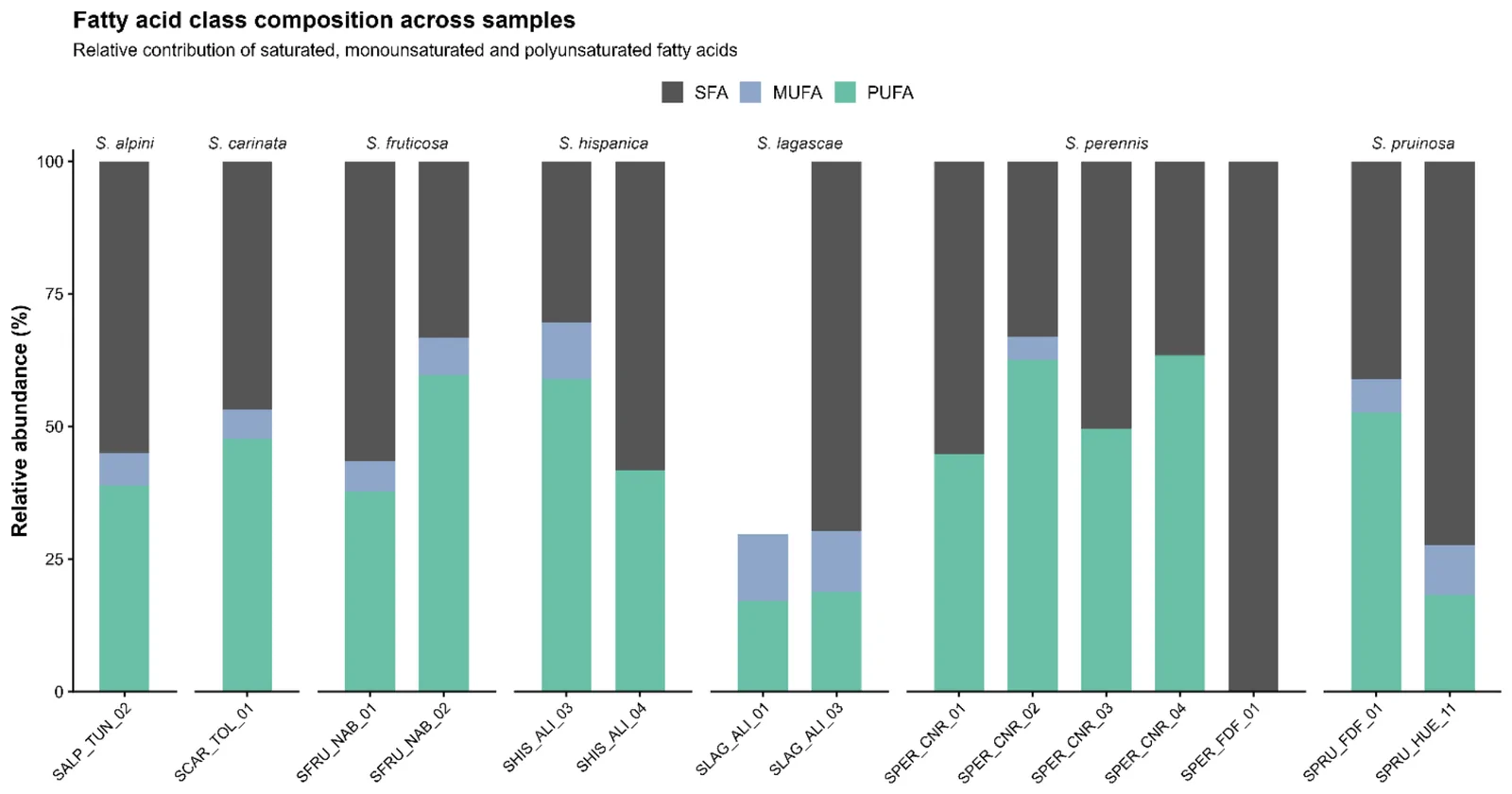
Relative composition of fatty acid classes across the studied localities. Saturated fatty acids (SFAs) included C12:0 (lauric acid), C14:0 (myristic acid), C16:0 (palmitic acid), C17:0 (margaric acid), C18:0 (stearic acid), C20:0 (arachidic acid), C22:0 (behenic acid), and C24:0 (lignoceric acid); monounsaturated fatty acids (MUFAs) included C18:1 (oleic acid); and polyunsaturated fatty acids (PUFAs) included C18:2 (linoleic acid) and C18:3 (α-linolenic acid). Each bar represents the percentage contribution of these three classes to the total fatty acid profile of a sample and sums to 100%.

The fatty acids identified in the analyzed perennial *Salicornia* species comprised saturated fatty acids (SFAs), monounsaturated fatty acids (MUFAs), and polyunsaturated fatty acids (PUFAs) (Figure 7).

Across the samples studied, lipid profiles were generally characterized by a predominance of SFAs or PUFAs, whereas MUFAs remained minor. *S. alpini* and *S. carinata* showed relatively similar intermediate profiles, although the proportion of PUFAs was higher in *S. carinata* (47.7%) than in *S. alpini* (38.8%). The clearest deviations from this general pattern were observed in *S. lagascae* from Santa Pola (SLAG_ALI_03), in which palmitic acid (C16:0) was the most abundant fatty acid, and *S. perennis* from Murraceira (SPER_FDF_01), which showed an exclusively saturated profile dominated by lignoceric acid (C24:0). Overall, the differences observed among localities highlighted marked intraspecific variation.

The most diverse class was the saturated fatty acids (SFAs), among which eight compounds were identified: lauric acid (C12:0), myristic acid (C14:0), palmitic acid (C16:0), margaric acid (C17:0), stearic acid (C18:0), arachidic acid (C20:0), behenic acid (C22:0), and lignoceric acid (C24:0). Among the monounsaturated fatty acids, only oleic acid (C18:1) was detected, and MUFAs never exceeded 12.5% (Figure 7). The polyunsaturated fatty acids identified were linoleic acid (C18:2) and α-linolenic acid (C18:3).

**Figure 8:**
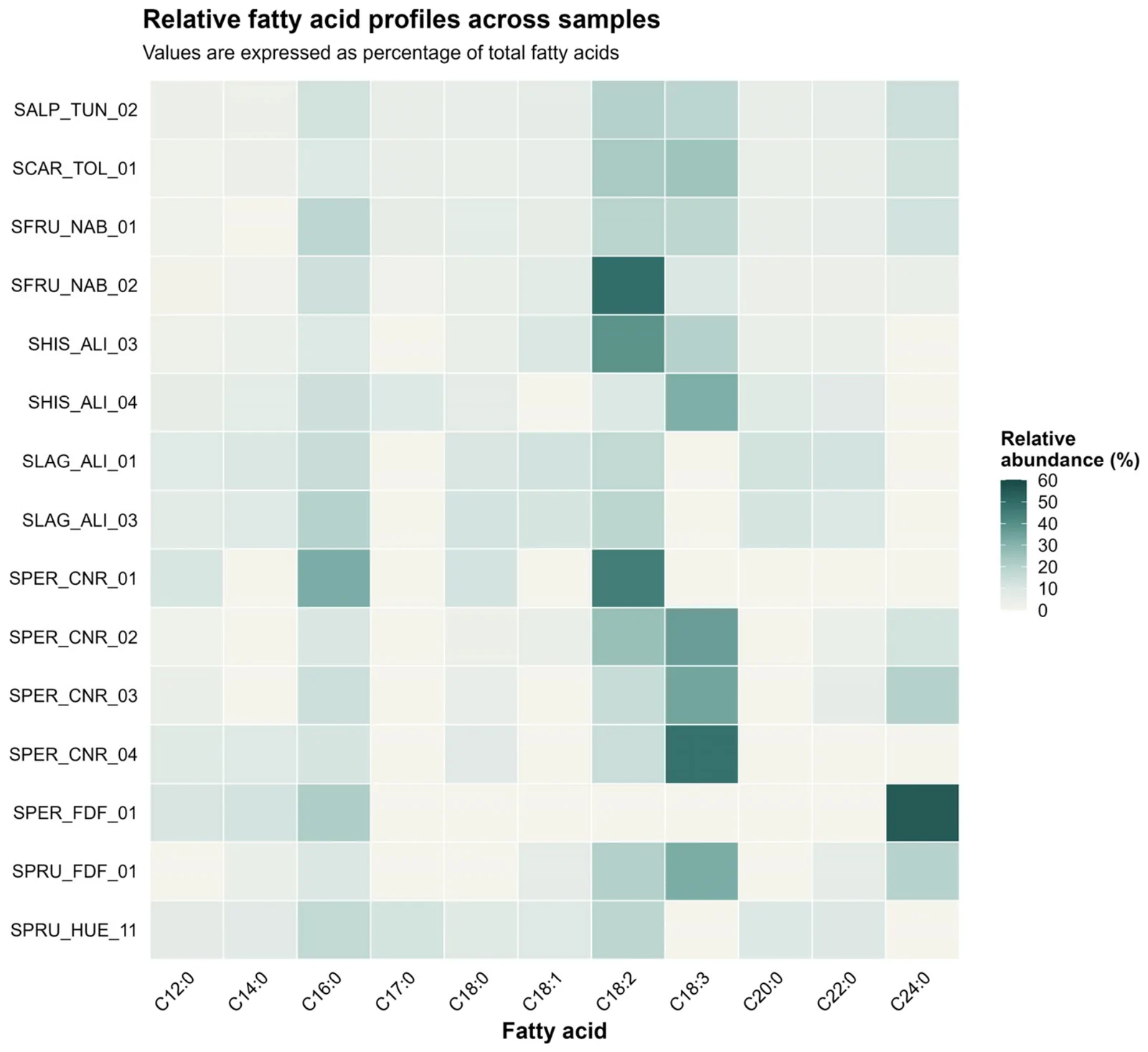
Relative fatty acid profile across the studied localities. The heatmap represents the abundance of C12:0 (lauric acid), C14:0 (myristic acid), C16:0 (palmitic acid), C17:0 (margaric acid), C18:0 (stearic acid), C18:1 (oleic acid), C18:2 (linoleic acid), C18:3 (α-linolenic acid), C20:0 (arachidic acid), C22:0 (behenic acid), and C24:0 (lignoceric acid), expressed as a percentage of the total fatty acid content of each sample. Darker shades indicate a greater relative contribution.

Palmitic acid (C16:0) was detected in all analyzed samples, whereas stearic acid (C18:0) and lauric acid (C12:0) were detected in 13 and 14 samples, respectively. These fatty acids were therefore among the most common constituents of the lipid profiles of the *Salicornia* species studied (Figure 8). On the other hand, margaric acid (C17:0) and arachidic acid (C20:0) were mainly detected in *S. alpini*, *S. carinata*, *S. fruticosa*, and *S. hispanica*. Arachidic acid was also detected in both populations of *S. lagascae*, whereas margaric acid was absent from this species. Neither compound was detected in the *S. perennis* samples.

In *S. pruinosa*, both fatty acids were absent from the Murraceira sample (SPRU_FDF_01) but present in the population from Isla Cristina (SPRU_HUE_11) (Figure 8).

Myristic acid (C14:0) and behenic acid (C22:0) were detected in most samples, occurring in 11 and 12 of the 15 samples, respectively, generally at low-to-moderate relative abundances. In contrast, lignoceric acid (C24:0) showed substantial variation among samples. Although it was absent from seven samples, it accounted for 55.0% of the total fatty acid profile in *S. perennis* from Murraceira (SPER_FDF_01), 20.3% in *S. perennis* from Lobos (SPER_CNR_03), and 19.9% in *S. pruinosa* from Murraceira (SPRU_FDF_01). This marked variation highlights the contribution of long-chain saturated fatty acids to the lipid profiles of some populations.

Regarding the polyunsaturated fatty acids, linoleic acid (C18:2) was detected in 14 of the 15 analyzed samples, whereas α-linolenic acid (C18:3) was present in 9 samples. When detected, both compounds frequently account for substantial proportions of the total fatty acid profile, with the highest relative abundances recorded for linoleic acid in *S. fruticosa* from Hammamet (SFRU_NAB_02; 49.4%) and for α-linolenic acid in *S. perennis* from La Santa (SPER_CNR_04; 48.6%). Their frequent occurrence and substantial relative contribution suggest a potential nutritional interest of the lipid fraction of the species studied.

**Figure 9:**
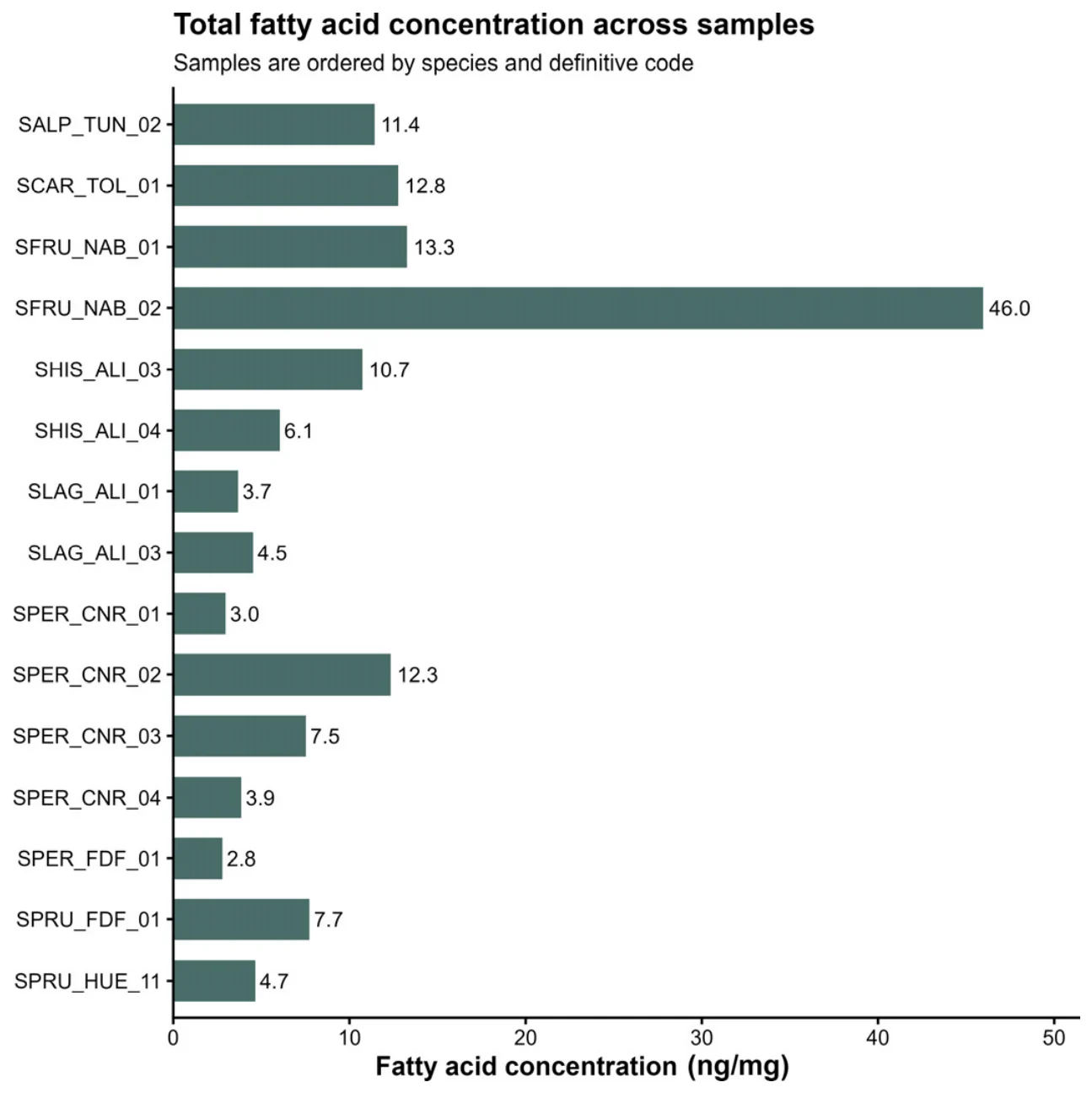
Total fatty acid concentration across the studied localities. Bars show the sum of the quantified fatty acids in each sample, grouped by species. Values are expressed as ng mg^−1^.

Total fatty acid concentration also varied widely. *S. fruticosa* from Hammamet (SFRU_NAB_02) reached 46.0 ng mg^−1^, more than three times the value recorded in Korba (SFRU_NAB_01; 13.3 ng mg^−1^). *S. carinata*, *S. perennis* from Órzola (SPER_CNR_02), and *S. alpini* (SALP_TUN_02) showed similar values, ranging from 11.4 to 12.8 ng mg^−1^. *S. hispanica* and *S. pruinosa* displayed intermediate concentrations, whereas *S. lagascae* showed low and relatively consistent values. The lowest concentrations were recorded in *S. perennis* from Murraceira (SPER_FDF_01) and Corralejo (SPER_CNR_01) at 2.8 and 3.0 ng mg^−1^, respectively (Figure 9).

The nutritional quality indices showed marked variation among populations (Table 9). The highest PUFA/SFA ratios were recorded in *S. hispanica* from El Hondo (1.94), *S. perennis* from Órzola (1.89), *S. fruticosa* from Hammamet (1.80), and *S. perennis* from La Santa (1.73). These populations also showed relatively low IA values (0.20–0.89) and high h/H ratios (2.20–5.01). In contrast, *S. lagascae* populations from Urbanova and Santa Pola exhibited the lowest PUFA/SFA ratios (0.24–0.27), the highest IA values (2.10–2.20), and the lowest h/H ratios (0.84–0.87). A similarly less favourable profile was observed in *S. pruinosa* from Isla Cristina. In *S. perennis* from Murraceira, PUFA and MUFA were not detected, resulting in a PUFA/SFA ratio and h/H ratio of zero, whereas IA could not be calculated.

## 4. Discussion

### 4.1. Elemental Composition

The elemental composition of the seven *Salicornia* species was dominated by Na, K, Mg, and Ca, with median concentrations of 72,132, 17,014, 16,560, and 8536 mg kg^−1^ DW, respectively. This pattern is consistent with the ion-accumulating strategy of succulent euhalophytes, in which Na plays an important role in osmotic adjustment, growth, and succulence [24]. Nevertheless, the relative abundance of Ca and Mg appears to vary among Salicornioideae. Mg exceeded Ca in our samples and in Portuguese material examined by Lopes et al. [10], whereas the opposite relationship was reported for Spanish *Salicornia* populations [25] and several Italian glassworts [26]. These observations suggest that the relative accumulation of divalent cations is not constant across taxa and growing environments.

Although the species exhibited different elemental concentration profiles, no statistically significant interspecific effects remained after correction for multiple testing. K, Ca, As, and Ba showed nominal species effects, but all four had FDR-adjusted *p* values of 0.084. Nevertheless, some descriptive patterns were consistent with previous observations. For example, the relatively low K and Ca concentrations in *S. pruinosa* (medians of 6734 and 4270 mg kg^−1^ DW, respectively) were comparable to those reported for populations from the Río Tinto salt marshes [16]. Other noteworthy observations included the elevated Fe and Ba concentrations in *S. perennis* and Ni concentrations in *S. alpini*. Previous studies have documented substantial variation in metal accumulation among Salicornioideae [27], as well as Fe biomineralization and intracellular Fe storage in *S. pruinosa* [16,28]. However, the small number of independent plants available for several species and the wide confidence intervals of some model estimates prevent these patterns from being interpreted as confirmed species-specific characteristics.

A particularly important feature of the present study was the substantial variability observed among individual plants and sampling locations. Unlike plants cultivated under controlled conditions, the specimens analyzed here originated from wild populations occupying diverse environments, including coastal salt marshes and inland endorheic saline lagoons, under Atlantic and Mediterranean climatic influences and on substrates of potentially different mineral composition. Variation in soil chemistry, salinity, flooding frequency, and nutrient availability could therefore contribute to the observed heterogeneity. Previous studies have demonstrated associations between environmental conditions and mineral accumulation in *S. carinata* [29] and other edible halophytes [26]. However, these environmental factors were not directly quantified in the present study, preventing their individual contributions from being established.

This environmental heterogeneity is particularly relevant to *S. pruinosa*, which exhibited markedly right-skewed distributions of As and Pb, including maximum concentrations of 181 and 51.9 mg kg^−1^ DW, respectively. Several sampled populations of this species originated from the Tinto–Odiel marsh system, an environment historically affected by metal-rich drainage and elevated concentrations of potentially toxic elements [27,30]. Exposure to metal-enriched sediments may have contributed to some of the elevated concentrations observed, although this interpretation remains hypothetical because corresponding soil and water chemistry data were unavailable. The variation observed within *S. pruinosa* emphasizes the potential importance of local substrate characteristics, element bioavailability, and plant uptake and compartmentalization processes. It also highlights the limitations of extrapolating elemental concentrations from individual wild populations to an entire species.

Phenological stage represented another potential source of variation. Although As, Sr, Ba, and Pb showed nominal associations with phenology, only As remained statistically significant after FDR correction. Fruiting plants exhibited an approximately 89% lower model-estimated As concentration than vegetative plants after adjustment for species identity (concentration ratio = 0.110; 95% CI: 0.032–0.377). This association was maintained in all leave-one-plant-out analyses but was no longer statistically significant when the comparison was restricted to species–site combinations containing both phenological stages. The limited within-site replication, incomplete representation of phenological stages across species, and concentration of non-detectable As measurements in *S. carinata* therefore constrain its interpretation. Although developmental variation in mineral accumulation has been documented in *Salicornia brachiata* [31], the present findings do not establish a direct physiological effect of phenological stage on As accumulation.

From a nutritional perspective, the mineral concentrations indicate potential value as food ingredients, although they must be considered alongside the high Na content. Using the overall arithmetic mean concentrations, 100 g of dried plant material would contain approximately 1627 mg K, 1684 mg Mg, 936 mg Ca, and 53.5 mg Fe. Compared with the European Food Safety Authority (EFSA) dietary reference values for adults, these amounts correspond to approximately 47% of the adequate intake (AI) for K, 481–561% of the AI for Mg, 94–99% of the population reference intake (PRI) for Ca, and 334–486% of the PRI for Fe, depending on age or sex [32]. Manganese and Cu could also contribute appreciably to dietary mineral intake, whereas Zn would provide a more modest contribution. However, the same 100 g of dried material would contain approximately 8.30 g Na, more than four times the EFSA safe and adequate adult intake of 2 g/day [33]. Species-specific comparisons also revealed differences in the balance between essential minerals and Na. *Salicornia carinata* showed a comparatively favorable Mg/Na ratio, together with a relatively high K/Na ratio, whereas *S. lagascae* exhibited the highest K/Na and Ca/Na ratios. Nevertheless, these descriptive patterns should not be interpreted as confirmed interspecific differences, and the elevated Na concentrations across all species remain an important nutritional limitation. Furthermore, these calculations are illustrative rather than serving-size recommendations, because actual nutritional contributions depend on consumption amounts, processing, mineral bioaccessibility, and the overall dietary context.

Food safety is therefore a critical consideration, particularly for material harvested from potentially contaminated environments. The maximum concentrations recorded for As, Pb, and Ni were 181, 51.9, and 21.3 mg kg^−1^ DW, respectively. For comparison, European Union legislation establishes maximum concentrations of 0.30 mg kg^−1^ for Pb and 0.50 mg kg^−1^ for Ni in the general category of leafy vegetables, expressed on a fresh-weight basis [34,35]. However, the applicability of these categories to the studied perennial *Salicornia* species and their potential food products requires confirmation. Furthermore, the absence of moisture measurements prevents direct conversion of the present dry-weight concentrations to fresh-weight equivalents. Consequently, regulatory compliance cannot be established from these data alone. Nevertheless, the elevated concentrations observed in certain plants warrant particular attention, particularly because of the health implications of chronic Pb exposure and dietary Ni exposure, including adverse reactions in Ni-sensitized individuals [30]. In addition, the present study measured total As without distinguishing inorganic from organic forms. Because inorganic As is of particular toxicological concern, chemical speciation would be necessary for a more specific dietary risk assessment [34].

Potentially excessive exposure to essential elements should also be considered when evaluating dried or concentrated plant products. The highest observed Mn and Cu concentrations (267 and 160 mg kg^−1^ DW, respectively) correspond to 26.7 and 16.0 mg per 100 g of dried material. These amounts exceed the EFSA adult safe intake level of 8 mg/day for Mn and the approximately 5 mg/day Cu intake considered unlikely to result in copper retention, respectively [36,37]. Importantly, EFSA has not established a tolerable upper intake level for Mn, and exceeding its safe intake level does not necessarily imply adverse health effects. Similarly, the concentrations of Sr and Ba, which reached 1077 and 108 mg kg^−1^ DW, respectively, suggest that these elements should also be considered when assessing exposure from concentrated plant products. However, these comparisons involve hypothetical consumption of material containing the highest observed concentrations, rather than established serving sizes or measured dietary exposures. They should therefore be interpreted as indications of potential exposure concerns, not evidence of toxicity.

Taken together, these findings highlight the need to evaluate both the nutritional potential and elemental safety of *Salicornia* before its use as food. The substantial variability observed among wild populations suggests that elemental composition may depend strongly on growing conditions, particularly substrate chemistry and element availability. Accordingly, food production should preferably rely on controlled cultivation systems with appropriate monitoring of soils or growing substrates and irrigation water, followed by systematic elemental quality control of harvested biomass and derived products [10,30]. Particular attention should be given to Na, As, Pb, and Ni, together with other elements that may reach elevated concentrations in dried or concentrated preparations. Such measures would help ensure more consistent mineral composition while minimizing exposure to potentially harmful elements.

### 4.2. Total Phenolic Content

Environmental conditions are another major source of variation in phenolic accumulation. Halophytes develop under multiple abiotic stresses, including high salinity, intense irradiation, and episodic water limitation, all of which can modify oxidative metabolism and stimulate the production of phenolic compounds involved in antioxidant protection [10]. In *Salicornia*, differences in phenolic content among populations have likewise been associated with contrasting environmental conditions, particularly salinity and exposure to other environmental stresses [38]. These responses occur together with broader metabolic adjustments, including changes in lipid metabolism. Accordingly, the presence of appreciable TPC values in all populations analyzed here is consistent with the recognized capacity of *Salicornia* to synthesize antioxidant phenolics and further supports its interest as a source of bioactive compounds with potential applications in functional foods.

The TPC values obtained in the present study were consistent with those previously reported for glassworts from the Iberian Peninsula. Annual *Salicornia* populations showed values ranging from 2.989 to 4.209 mg GAE/g DW [39], while perennial species previously treated in the former recognized *Sarcocornia* showed values between 3.231 and 3.892 mg GAE/g DW [38]. The range recorded here, 2.579–3.924 mg GAE/g DW, largely overlapped these earlier findings.

TPC values were relatively similar across the species and populations examined. Particularly close values were recorded between the two populations of *S. lagascae*, *S. fruticosa*, and *S. hispanica*.

These results expand the available information on the phenolic content of perennial *Salicornia*. Given the recognized contribution of phenolic compounds to the antioxidant properties of halophytes, the concentrations recorded support the potential of perennial *Salicornia* as a source of bioactive compounds [40,41,42].

### 4.3. Phenolic Compounds

A total of 18 phenolic compounds were quantified in the analyzed plant material, including hydroxybenzoic and hydroxycinnamic acids, flavonoids, the lignan pinoresinol, and hydroxytyrosol. Chlorogenic acid and rutin were detected in all samples, whereas the other phenolic acids and flavonoids showed greater variation in occurrence and concentration among taxa and populations. This broad profile is consistent with previous studies of annual and perennial *Salicornia* species [10,38,39,42,43].

Among the phenolic acids, ferulic and chlorogenic acids showed the most relevant concentrations in the present study. Ferulic acid was detected in all analyzed samples, extending previous reports in which it was identified only in particular halophytic taxa, *Arthrocaulon macrostachyum*, a genus closely related to *Salicornia* [38]. Its widespread occurrence is noteworthy because ferulic acid has been associated with antioxidant defense and cell-wall reinforcement in plants. In *S. europaea*, salinity altered phenylpropanoid metabolism and significantly increased ferulic acid concentrations in the roots [44]. This response may provide a physiological context for the frequent occurrence of ferulic acid in the perennial *Salicornia* taxa analyzed here, although its specific role in these species remains to be established.

Chlorogenic acid was also detected across the analyzed taxa and reached its highest concentrations in *S. perennis* from Lanzarote and Fuerteventura (Canary Island, Spain). Comparable values occurred in some Atlantic and Mediterranean populations of *S. pruinosa*, *S. hispanica*, *S. carinata*, and *S. fruticosa*, although the concentrations were generally low. Chlorogenic acid has previously been reported on annual *Salicornia* species and may contribute to their antioxidant capacity [43,44]. Its concentration can nevertheless vary according to tissue, developmental stage, salinity, and geographic origin. The occurrence of chlorogenic acid in the perennial *Salicornia* taxa examined here therefore extends the information available for this group, particularly for the less frequently studied species.

The flavonoid profile was dominated by quercetin derivatives. Isoquercetin was detected in all samples and reached its highest concentrations in *S. carinata*, *S. alpini*, and *S. fruticosa*, whereas lower values were generally observed in the other analyzed taxa, including *S. perennis*, *S. pruinosa*, *S. hispanica*, and *S. lagascae*. Rutin was also detected in all samples, although its concentration varied considerably, while free quercetin occurred at much lower levels. Apigenin, apigenin-7-O-glucoside, luteolin, and luteolin-7-O-glucoside were detected only sporadically. The widespread occurrence of rutin and isoquercetin agrees with previous reports identifying quercetin and its glycosylated derivatives as recurrent constituents of annual and perennial *Salicornia* [4,38,39,43,45,46]. Their greater abundance than free quercetin is consistent with the predominance of glycosylated forms in the analyzed tissues.

The seeds analyzed in the present study contained salicylic, ferulic, and chlorogenic acids, together with the flavonoids quercetin, rutin, and isoquercetin. Rutin and isoquercetin were the predominant compounds and together accounted for 65.1–96.2% of the total quantified content, although their relative contributions varied among taxa and populations. Previous studies on annual *Salicornia* seeds have mainly focused on total phenolic and flavonoid contents, antioxidant and cytotoxic activities, or seed-oil composition [11,47,48,49]. In contrast, the present study provides information on individual phenolic acids and flavonoids in the seeds of perennial *Salicornia* taxa. To the best of our knowledge, this is the first report of these individual phenolic acids in the seeds of the taxa investigated here.

### 4.4. Fatty Acids

In the present study, eleven fatty acids were identified in the analyzed *Salicornia* species, including saturated fatty acids (SFAs), monounsaturated fatty acids (MUFAs), and polyunsaturated fatty acids (PUFAs).

Recent studies on halophytes support the view that variation in lipid profiles may form part of the physiological response to environmental stress. In *Salicornia europaea*, salt exposure has been associated with substantial remodeling of lipid metabolism and changes in membrane lipid composition that may contribute to membrane stability and ionic homeostasis under saline conditions [50]. Likewise, variation in fatty acid composition has been observed under different cultivation and environmental conditions in *Salicornia* species [51,52].

Palmitic acid (C16:0) was detected in all analyzed samples and was one of the most consistently occurring saturated fatty acids, in agreement with previous studies reporting C16:0 as a major saturated fatty acid in several species of the genus [3,32]. In addition to its previously reported occurrence in *S. perennis* and *S. alpini*, the present study confirms its presence in *S. carinata*, *S. fruticosa*, *S. hispanica*, and *S. lagascae*. The highest concentration was recorded in *S. fruticosa* (6.16 ng mg^−1^). Likewise, lauric acid (C12:0) and stearic acid (C18:0) were detected in 14 and 13 of the 15 samples analyzed, respectively, indicating that these saturated fatty acids are common components of the lipid profile of the species examined.

Interestingly, margaric acid (C17:0) was detected in several *Salicornia* species inhabiting Mediterranean coastal salt marshes and inland saline habitats, including *S. alpini*, *S. carinata*, *S. fruticosa*, and *S. hispanica*, as well as in one population of *S. pruinosa*. Previous studies had already reported this fatty acid in *S. perennis* subsp. *perennis* and *S. perennis* subsp. *alpini* from Portuguese populations [4,45]. In the present study, however, margaric acid was absent from all analyzed populations of *S. perennis*. These findings suggest that the occurrence and abundance of margaric acid may vary among species and populations and may also be influenced by environmental conditions characteristic of different saline habitats.

Among the monounsaturated fatty acids, oleic acid (C18:1) was identified, whereas palmitoleic acid (C16:1) was not detected in any of the analyzed *Salicornia* samples. This contrasts with previous reports from Portuguese populations of *S. perennis* and *S. alpini*, in which palmitoleic acid was identified [4,45]. Differences in fatty acid composition among populations may be influenced by species-specific responses and environmental conditions. In *Salicornia ramosissima*, for example, fatty acid profiles have been shown to vary in response to temperature and CO_2_ conditions, although no significant changes attributable to salinity or drought were detected in that study [52]. Cultivation conditions may also modify lipid composition [51].

Several analyzed samples exhibited high proportions of polyunsaturated fatty acids (PUFAs), particularly linoleic acid (C18:2) and α-linolenic acid (C18:3). High concentrations of both fatty acids were recorded in *S. carinata*, *S. fruticosa*, and *S. perennis*. Notably, *S. hispanica*, especially the population collected at San Felipe Neri, showed elevated concentrations of linoleic acid (4.16 ng mg^−1^) and α-linolenic acid (2.17 ng mg^−1^). Changes in the degree of fatty acid unsaturation are considered part of the adaptive response of halophytes to saline environments because they can contribute to maintaining membrane properties under stress conditions [38,50,53].

Beyond their physiological role in plants, polyunsaturated fatty acids are bioactive compounds of considerable nutritional interest. Previous studies on *Salicornia* have highlighted the nutritional relevance of lipid profiles rich in unsaturated fatty acids and their potentially favorable atherogenic and thrombogenic indices [4]. Other studies have also associated fatty acids identified in these plants with different biological activities [38].

The three indices revealed clear population-level differences in lipid quality. *S. hispanica* from El Hondo, *S. fruticosa* from Hammamet, and *S. perennis* from Órzola and La Santa showed particularly favourable profiles, combining high PUFA/SFA and h/H values with low IA values. These patterns were associated with a substantial contribution of linoleic and α-linolenic acids and highlight the nutritional interest of selected perennial *Salicornia* populations, in agreement with previous findings for the edible halophyte *S. ramosissima* [54].

Therefore, the substantial contribution of PUFAs, particularly linoleic and α-linolenic acids, observed in several *Salicornia* species further supports their potential as functional foods and as valuable sources of nutritionally relevant bioactive compounds.

## 5. Conclusions

This study provides a comparative characterization of the mineral and bioactive composition of seven perennial *Salicornia* species across multiple populations and environments. A common elemental profile dominated by Na, K, Mg, and Ca was accompanied by species-related differences in K, Ca, Fe, Ni, and Ba and by phenology-related variation in several trace elements, showing that perennial *Salicornia* cannot be represented by a single elemental profile.

The lipid quality indices based on the PUFA/SFA ratio, the atherogenicity index (AI), and the hypocholesterolemic/hypercholesterolemic ratio (h/H) revealed highly favorable lipid profiles in the perennial *Salicornia* species, particularly *S. hispanica*, *S. fruticosa*, and *S. perennis*. These patterns were associated with a substantial contribution of linoleic and α-linolenic acids, highlighting the nutritional potential of perennial *Salicornia* populations.

The analysis of phenolic compounds revealed moderate total contents but marked variation in the individual compounds. Several glycosylated flavonoids were identified. Isoquercetin and rutin were the predominant forms in the aerial tissues, whereas luteolin-7-O-glucoside and apigenin-7-O-glucoside occurred only sporadically. Rutin and isoquercetin were also the predominant phenolic compounds among those quantified in the seeds.

A key contribution of this study is the inclusion of data on the fatty acid, phenolic compound, and flavonoid contents of perennial *Salicornia* species, namely *S. alpini*, *S. carinata*, *S. fruticosa*, *S. hispanica*, *S. lagascae*, *S. perennis*, and *S. pruinosa*. Given the scarcity of available data on phenolic compounds and flavonoids in the seeds of annual *Salicornia* species, our study provides, for the first time, data on these compounds in the seeds of these seven perennial species.

## Figures and Tables

**Table 1:** Collection information of each sample used in this study: code, origin (country, province and specific site) and WGS84 coordinates.

Code	Species	Country	Province	Collection Information	Coordinates WGS84 (Lat, Lon)
SALP_HUE_01	*S. alpini*	Spain	Huelva	La Rábida	37.213250, −6.914910
SALP_HUE_02	*S. alpini*	Spain	Huelva	San Juan Puerto	37.314220, −6.840780
SALP_SEV_01	*S. alpini*	Spain	Sevilla	Doñana, C. J.A.Valverde	37.073610, −6.376720
SALP_TUN_01	*S. alpini*	Tunisia	Nabeul	Yasmine Hammamet	36.374690, 10.541150
SALP_TUN_02	*S. alpini*	Tunisia	Tunis	Gammarth, Lac de Tunis	36.823700, 10.21100
SCAR_TOL_01	*S. carinata*	Spain	Toledo	Laguna larga de Villacañas	39.603380, −3.316700
SCAR_TOL_02	*S. carinata*	Spain	Toledo	Laguna de Peñahueca	39.514050, −3.340270
SCAR_TOL_03	*S. carinata*	Spain	Toledo	Laguna de Peñahueca	39.514050, −3.340270
SCAR_TOL_04	*S. carinata*	Spain	Toledo	Laguna de Peñahueca	39.514050, −3.340270
SCAR_TOL_05	*S. carinata*	Spain	Toledo	Laguna de Peñahueca	39.514050, −3.340270
SCAR_TOL_06	*S. carinata*	Spain	Toledo	Laguna Larga de Villacañas	39.603380, −3.316700
SCAR_TOL_07	*S. carinata*	Spain	Toledo	Laguna Larga de Villacañas	39.603380, −3.316700
SCAR_TOL_08	*S. carinata*	Spain	Toledo	Laguna Larga de Villacañas	39.603380, −3.316700
SCAR_TOL_09	*S. carinata*	Spain	Toledo	Laguna Larga de Villacañas	39.603380, −3.316700
SCAR_TOL_10	*S. carinata*	Spain	Toledo	Laguna larga Villacañas	39.603380, −3.316700
SCAR_TOL_11	*S. carinata*	Spain	Toledo	Laguna Tirez	39.535240, −3.357430
SFRU_SAR_01	*S. fruticosa*	Italy	Sardinia	Sta. Gilla, Cagliari, P. Vecchio	39.216390, 9.044170
SFRU_FRA_01	*S. fruticosa*	France	Occitania	Frontignan	43.447200, 3.755600
SFRU_NAB_01	*S. fruticosa*	Tunisia	Nabeul	Korba	36.572890, 10.857620
SFRU_NAB_02	*S. fruticosa*	Tunisia	Nabeul	Yasmine Hammamet dunes	36.374690, 10.541150
SHIS_ALI_01	*S. hispanica*	Spain	Alicante	San Felipe Neri, El Hondo	38.177680, −0.796830
SHIS_ALI_02	*S. hispanica*	Spain	Alicante	San Felipe Neri, El Hondo	38.177680, −0.796830
SHIS_ALI_03	*S. hispanica*	Spain	Alicante	Villena, Las Virtudes	38.625690, −0.931970
SHIS_ALI_04	*S. hispanica*	Spain	Alicante	El Hondo	38.183330, −0.753060
SHIS_ALI_05	*S. hispanica*	Spain	Alicante	Sta. Pola, S. del Bras del Port	38.202000, −0.578860
SHIS_ALB_01	*S. hispanica*	Spain	Albacete	Cordovilla	38.564770, −1.638350
SLAG_ALI_01	*S. lagascae*	Spain	Alicante	Urbanova	38.283490, −0.521730
SLAG_MEN_01	*S. lagascae*	Spain	Menorca	Albufera des Grau	39.940080, 4.251680
SLAG_ALI_02	*S. lagascae*	Spain	Alicante	Sta Pola, La Gola	38.191950, −0.522640
SLAG_ALI_03	*S. lagascae*	Spain	Alicante	Sta. Pola, S. del Bras del Port	38.202000, −0.578860
SPER_TAV_01	*S. perennis*	Portugal	Tavira	Tavira salt marshes	37.070000, −7.680000
SPER_FDF_01	*S. perennis*	Portugal	Figueira da Foz	Isla da Murraceira	40.116670, −8.816670
SPER_CNR_01	*S. perennis*	Spain	Canary Island	Corralejo, Charco del Bristol	28.740100, −13.869300
SPER_CNR_02	*S. perennis*	Spain	Canary Island	Haría, Órzola, Caletón Blanco	29.222900, −13.452200
SPER_CNR_03	*S. perennis*	Spain	Canary Island	La Oliva, isla de Lobos	28.750220, −13.820520
SPER_CNR_04	*S. perennis*	Spain	Canary Island	Tinajo, La Santa Sport	29.107730, −13.665680
SPER_CTB_01	*S. perennis*	Spain	Cantabria	Ría del Capitán, Oyambre	43.397600, −4.341350
SPER_HUE_01	*S. perennis*	Spain	Huelva	Isla Cristina	37.203700, −7.323900
SPRU_HUE_01	*S. pruinosa*	Spain	Huelva	Isla Cristina	37.203700, −7.323900
SPRU_FDF_01	*S. pruinosa*	Portugal	Figueira da Foz	Isla da Murraceira	40.116670, −8.816670
SPRU_HUE_02	*S. pruinosa*	Spain	Huelva	La Rabida	37.213250, −6.914910
SPRU_HUE_03	*S. pruinosa*	Spain	Huelva	La Rábida	37.213250, −6.914910
SPRU_HUE_04	*S. pruinosa*	Spain	Huelva	La Rábida	37.213250, −6.914910
SPRU_HUE_05	*S. pruinosa*	Spain	Huelva	Moguer	37.274620, −6.837350
SPRU_HUE_06	*S. pruinosa*	Spain	Huelva	Monumento a Colón	37.212300, −6.940370
SPRU_HUE_07	*S. pruinosa*	Spain	Huelva	San Juan del Puerto	37.314220, −6.840780
SPRU_HUE_08	*S. pruinosa*	Spain	Huelva	Tinto river estuary	37.212300, −6.940370
SPRU_HUE_09	*S. pruinosa*	Spain	Huelva	La Rabida	37.213250, −6.914910
SPRU_HUE_10	*S. pruinosa*	Spain	Huelva	San Juan del Puerto	37.314220, −6.840780
SPRU_HUE_11	*S. pruinosa*	Spain	Huelva	Isla Cristina, Marsh Foods	37.203700, −7.323900
SPRU_HUE_12	*S. pruinosa*	Spain	Huelva	Isla Cristina, Marsh Foods	37.203700, −7.323900
SPRU_SAN_01	*S. pruinosa*	Spain	Santander	Santoña saltmarshes	43.437300, −3.480400

**Table 2:** Sampling structure of the *Salicornia* dataset. Number of independent plants, analytical portions, and sampling sites for each species, together with the distribution of plants between vegetative and fruiting stages. When multiple portions were available for an individual plant, elemental concentrations were averaged on the original scale before statistical analysis. Two sampling sites (La Rábida and San Juan del Puerto) were shared by *S. alpini* and *S. pruinosa*. Phenological counts refer to independent plants rather than analytical portions.

Species	Plants	Analytical Portions	Sampling Sites	Vegetative	Fruiting
*S. carinata*	24	24	2	18	6
*S. perennis*	2	5	2	2	0
*S. pruinosa*	8	12	6	4	4
*S. alpini*	4	10	4	1	3
*S. hispanica*	4	6	2	1	3
*S. lagascae*	2	6	2	0	2
*S. fruticosa*	2	5	2	1	1
Total	46	68	18	27	19

**Table 3:** Overall elemental composition of the studied *Salicornia* samples. Element concentrations are expressed as mg kg^−1^ dry weight (DW) and were summarized at the plant level. For plants represented by multiple analytical portions, concentrations were first averaged on the original scale so that each plant contributed a single independent observation. Values are reported as the arithmetic mean ± standard deviation (SD), median, and minimum–maximum range. *n* indicates the number of plants with a valid measurement for each element.

Element	*n* Valid	Mean ± SD	Median	Range
Na	46	82,977 ± 56,470	72,132	9527–366,794
Mg	46	16,842 ± 8326	16,560	298–41,735
K	46	16,274 ± 7760	17,014	743–38,221
Ca	46	9361 ± 5870	8536	2583–34,954
Mn	46	41.1 ± 39.8	30.3	7.30–267
Fe	46	535 ± 1047	230	37.2–5814
Ni	46	3.60 ± 4.84	1.67	0.280–21.3
Cu	46	13.7 ± 24.8	6.59	0.910–160
Zn	46	29.4 ± 31.5	20.3	5.61–186
As	30	10.0 ± 34.4	0.425	0.020–181
Sr	46	151 ± 239	74.2	0.020–1077
Ba	46	7.93 ± 16.7	2.85	0.520–108
Pb	45	3.21 ± 8.20	0.553	0.050–51.9

**Table 4:** Species-specific descriptive statistics for elemental concentrations in the seven *Salicornia* species. Values are presented as arithmetic mean ± standard deviation (SD) and median and are expressed as mg kg^−1^ dry weight. Non-detects were excluded on an element-by-element basis; consequently, sample sizes for As, Sr and Pb may be lower than the total number of samples available for each species.

Element	*S. alpini*		*S. carinata*		*S. fruticosa*		*S. hispanica*		*S. lagascae*		*S. perennis*		*S. pruinosa*	
	Mean ± SD	Median	Mean ± SD	Median	Mean ± SD	Median	Mean ± SD	Median	Mean ± SD	Median	Mean ± SD	Median	Mean ± SD	Median
Na	93,198 ± 40,341	77,230	69,633 ± 17,803	68,346	120,878 ± 77,582	120,878	139,269 ± 152,865	76,490	120,768 ± 53,567	120,768	146,909 ± 84,162	146,909	54,845 ± 33,061	49,173
K	16,126 ± 10,678	19,387	18,524 ± 4785	18,897	20,604 ± 1269	20,604	12,376 ± 7898	10,833	32,448 ± 8164	32,448	15,977 ± 5153	15,977	6497 ± 2700	6734
Mg	11,017 ± 2362	10,004	21,659 ± 6316	20,158	12,536 ± 5395	12,536	19,590 ± 8757	16,653	16,732 ± 1105	16,732	15,961 ± 6546	15,961	5250 ± 2556	5545
Ca	11,296 ± 5956	9679	7735 ± 2349	8497	13,699 ± 3658	13,699	14,642 ± 4787	14,165	22,321 ± 17,866	22,321	16,645 ± 6882	16,645	4484 ± 1102	4270
Fe	555 ± 174	536	204 ± 126	187	405 ± 14.5	405	249 ± 184	249	761 ± 755	761	4967 ± 1198	4967	530 ± 791	222
Mn	41.5 ± 28.0	39.7	38.0 ± 13.2	35.6	28.8 ± 2.78	28.8	20.6 ± 2.09	20.6	24.0 ± 8.84	24.0	95.7 ± 26.0	95.7	54.2 ± 87.7	22.1
Cu	19.9 ± 12.4	18.1	6.43 ± 2.63	5.36	9.29 ± 2.14	9.29	17.5 ± 15.5	16.8	9.03 ± 0.209	9.03	13.6 ± 9.60	13.6	32.8 ± 55.7	6.91
Ni	11.1 ± 7.88	10.3	1.02 ± 0.517	0.860	8.08 ± 5.22	8.08	2.65 ± 1.44	2.43	3.29 ± 1.95	3.29	10.6 ± 10.6	10.6	5.26 ± 4.79	4.57
Zn	33.3 ± 10.1	36.7	15.3 ± 6.95	16.2	31.7 ± 2.47	31.7	30.7 ± 16.1	31.5	27.6 ± 17.9	27.6	40.7 ± 11.2	40.7	66.4 ± 60.9	43.0
Sr	102 ± 117	53.5	74.4 ± 38.4	74.2	75.7 ± 53.3	75.7	380 ± 465	160	659 ± 445	659	547 ± 505	547	81.3 ± 146	43.7
Ba	12.7 ± 8.36	10.6	2.52 ± 1.84	2.08	5.16 ± 0.764	5.16	2.67 ± 2.23	2.22	10.9 ± 11.2	10.9	70.7 ± 53.4	70.7	8.68 ± 7.35	7.69
As	0.823 ± 0.569	0.591	0.174 ± 0.103	0.160	1.24 ± 1.17	1.24	1.19 ± 1.30	0.769	0.403 ± 0.359	0.403	4.21 ± 0.581	4.21	34.9 ± 62.6	4.14
Pb	1.56 ± 0.754	1.39	0.339 ± 0.274	0.270	1.86 ± 2.28	1.86	2.62 ± 2.89	2.41	1.45 ± 1.50	1.45	5.02 ± 2.28	5.02	12.9 ± 16.7	8.00

**Table 5:** Global tests of interspecific differences in elemental concentrations among *Salicornia* species. Elemental concentrations were analyzed after log_10_ transformation using linear mixed-effects models with species as a fixed effect and sampling site as a random intercept. For As, the sampling-site variance collapsed to zero; therefore, a linear model including species as the fixed effect was used. For mixed-effects models, denominator degrees of freedom and *F* tests were obtained using the Satterthwaite approximation. Reported *p* values correspond to the global species effect for each element. False discovery rate (FDR)-adjusted *p* values were calculated across the 13 elemental tests using the Benjamini–Hochberg procedure. LMM, linear mixed-effects model; LM, linear model; FDR, false discovery rate.

Element	Model	n	F (df1, df2)	*p*-Value	FDR-Adjusted *p*
Na	LMM	46	1.26 (6, 9.07)	0.361	0.469
Mg	LMM	46	2.33 (6, 12.13)	0.099	0.185
K	LMM	46	3.85 (6, 12.45)	0.021	0.084
Ca	LMM	46	6.90 (6, 4.98)	0.026	0.084
Mn	LMM	46	0.74 (6, 10.62)	0.629	0.681
Fe	LMM	46	4.58 (6, 4.28)	0.074	0.177
Ni	LMM	46	2.62 (6, 10.60)	0.082	0.177
Cu	LMM	46	0.41 (6, 12.00)	0.856	0.856
Zn	LMM	46	1.07 (6, 9.95)	0.442	0.522
As	LM	30	3.34 (6, 23.00)	0.016	0.084
Sr	LMM	46	1.33 (6, 11.08)	0.322	0.465
Ba	LMM	46	7.25 (6, 7.33)	0.009	0.084
Pb	LMM	45	5.87 (6, 2.00)	0.153	0.248

**Table 6:** Associations between phenological stage and elemental concentrations in the seven studied *Salicornia* species. Elemental concentrations (mg kg^−1^ dry weight, DW) were analyzed at the individual plant level after log_10_ transformation. Linear mixed-effects models (LMMs) included species and phenological stage as fixed effects and sampling site as a random intercept. Arsenic (As) was analyzed using a linear model (LM) because the estimated sampling-site variance was zero. Phenological effects are expressed as back-transformed concentration ratios between fruiting and vegetative plants (Fruit/Vegetative), with corresponding 95% confidence intervals (CIs). Ratios greater than 1 indicate higher model-estimated concentrations in fruiting plants, whereas ratios below 1 indicate lower concentrations. Degrees of freedom for LMMs were estimated using the Satterthwaite approximation. *p*-values were adjusted across the 13 elemental tests using the Benjamini–Hochberg false discovery rate (FDR) procedure. Confidence intervals were not adjusted for multiple comparisons. Sample sizes (n) represent independent plants with valid measurements for each element. Only As, in bolds, remained statistically significant after FDR correction (adjusted *p* < 0.05).

Element	Model	n	Concentration Ratio (Fruit/Veg)	95% CI	*p*-Value	FDR-Adjusted *p*
Na	LMM	46	0.921	0.675–1.257	0.5931	0.5931
Mg	LMM	46	0.925	0.740–1.157	0.4813	0.5214
K	LMM	46	1.180	0.934–1.489	0.1578	0.2564
Ca	LMM	46	0.871	0.680–1.115	0.2631	0.3800
Mn	LMM	46	1.289	0.913–1.819	0.1431	0.2564
Fe	LMM	46	0.634	0.394–1.018	0.0589	0.1532
Ni	LMM	46	1.414	0.892–2.241	0.1350	0.2564
Cu	LMM	46	1.252	0.762–2.056	0.3634	0.4485
Zn	LMM	46	0.848	0.581–1.236	0.3795	0.4485
**As**	**LM**	**30**	**0.110**	**0.032–0.377**	**0.0012**	**0.0157**
Sr	LMM	46	0.423	0.204–0.876	0.0221	0.0718
Ba	LMM	46	0.488	0.286–0.832	0.0101	0.0629
Pb	LMM	45	0.409	0.202–0.827	0.0145	0.0629

**Table 7:** Total phenolic content (TPC; mg GAE/g DW) in perennial *Salicornia* samples. Values are means ± SD.

Species	Sample Code	TPC ± SD
*S. alpini*	SALP_TUN_02	3.419 ± 0.074
*S. carinata*	SCAR_TOL_01	3.488 ± 0.105
*S. fruticosa*	SFRU_NAB_01	3.433 ± 0.055
*S. fruticosa*	SFRU_NAB_02	3.503 ± 0.102
*S. hispanica*	SHIS_ALI_03	3.816 ± 0.082
*S. hispanica*	SHIS_ALI_04	3.924 ± 0.052
*S. lagascae*	SLAG_ALI_01	2.583 ± 0.008
*S. lagascae*	SLAG_ALI_03	2.579 ± 0.059
*S. pruinosa*	SPRU_HUE_11	3.705 ± 0.077

**Table 8:** Total concentration and relative composition of the six phenolic compounds quantified in seeds.

Sample	Total (ng/mg)	Rutin (%)	Isoquercetin (%)	Quercetin (%)	Rutin + Isoquercetin (%)	Salicylic Acid (%)	Ferulic Acid (%)	Chlorogenic Acid (%)
SALP_TUN_01	10,201	44.5	51.7	0.8	96.2	0.0	2.3	0.7
SCAR_TOL_10	9004	39.9	54.6	3.1	94.5	0.0	1.5	0.9
SCAR_TOL_11	5994	67.6	15.8	1.3	83.4	0.1	9.1	6.2
SFRU_SAR_01	10,790	61.6	31.9	1.5	93.4	0.0	3.9	1.2
SLAG_MEN_01	7140	42.3	51.9	0.7	94.2	0.1	4.1	1.0
SPER_HUE_01	3155	82.2	6.6	0.0	88.8	0.0	9.2	2.0
SPRU_HUE_01	1638	64.3	14.6	0.5	78.9	0.2	13.2	7.2
SPRU_HUE_08	1618	59.0	6.0	1.2	65.1	1.4	31.8	0.5
SPRU_HUE_09	3588	58.5	29.6	2.2	88.1	0.6	8.6	0.6
SPRU_HUE_12	1812	60.2	22.7	0.0	82.9	0.2	2.1	14.8
SPRU_SAN_01	1963	60.5	22.5	1.1	82.9	0.0	12.2	3.8

**Table 9:** Nutritional quality indices of fatty acids across the studied populations.

Species	Population	Sample Code	PUFA/SFA	IA	h/H
*S. alpini*	Salloum	SALP_TUN_02	0.71	0.64	2.37
*S. carinata*	Villacañas	SCAR_TOL_01	1.02	0.44	3.57
*S. fruticosa*	Korba	SFRU_NAB_01	0.67	0.48	2.11
*S. fruticosa*	Hammamet	SFRU_NAB_02	1.8	0.3	4.24
*S. hispanica*	El Hondo	SHIS_ALI_03	1.94	0.39	4.27
*S. hispanica*	Villena	SHIS_ALI_04	0.71	1.11	1.58
*S. lagascae*	Urbanova	SLAG_ALI_01	0.24	2.2	0.87
*S. lagascae*	Santa Pola	SLAG_ALI_03	0.27	2.1	0.84
*S. perennis*	Corralejo	SPER_CNR_01	0.81	0.96	1.04
*S. perennis*	Órzola	SPER_CNR_02	1.89	0.2	5.01
*S. perennis*	Lobos	SPER_CNR_03	0.98	0.38	2.66
*S. perennis*	La Santa	SPER_CNR_04	1.73	0.89	2.2
*S. perennis*	Murraceira	SPER_FDF_01	0	NA	0
*S. pruinosa*	Murraceira	SPRU_FDF_01	1.28	0.49	3.89
*S. pruinosa*	Isla Cristina	SPRU_HUE_11	0.25	2.03	0.86

Footnote: PUFA/SFA, ratio of polyunsaturated to saturated fatty acids; IA, index of atherogenicity; h/H, hypocholesterolemic/hypercholesterolemic ratio. Higher PUFA/SFA and h/H values and lower IA values generally indicate a more favourable fatty-acid profile. IA could not be calculated for *S. perennis* from Murraceira because MUFA and PUFA were not detected.

## Data Availability

The original contributions presented in this study are included in the article. Further inquiries can be directed to the corresponding authors.

## Abbreviations

The following abbreviations are used in this manuscript:

DW	Dry weight
MUFAs	Monounsaturated fatty acids
PUFAs	Polyunsaturated fatty acids
POWO	Plants of the World Online
SFAs	Saturated fatty acids
TPC	Total Phenolic Content

## Appendix A

**Table A1:** Enumeration of authentic trading patterns treated. For each analyte, the precursor-to-product ion transitions, collision energies (CE), and retention times of the phenolic compounds analyzed by HPLC-ESI-MS/MS.

Compound	Precursor Ion	Product Ion	Collision Energy	Retention Time (min)
Gallic acid	169	125	9	2.71
	169	79	20	
Hydroxytyrosol	153	123	10	3.65
	153	95	16	
Clorogenic acid	353	191	9	3.87
	353	85	28	
	353	93	31	
Tyrosol	137	106	11	4.32
	137	119	10	
	137	93	14	
Caffeic acid	179	135	12	4.57
Syringic acid	197	123	20	4.60
	197	182	8	
	197	153	8	
	197	138	13	
Rutin	609	300	28	4.61
	609	271	56	
Vanilic acid	167	152	8	4.66
	167	108	14	
	167	123	7	
Luteolin-7-O-glucoside	447	285	23	4.86
Isoquercetin	463	300	21	4.87
	463	271	38	
	463	255	36	
Apigenin-7-glucoside	431	268	31	5.25
	431	239	49	
Sinapic acid	223	149	17	5.30
	223	164	8	
	223	208	7	
Ferulic acid	193	134	10	5.43
	193	178	9	
	193	149	6	
Oleuropeina	539	307	15	5.48
	539	275	18	
	539	377	7	
	539	139	26	
Cumaric acid	163	119	9	5.62
	163	91	21	
Salicylic acid	137	93	10	6.06
	137	65	24	
	137	75	30	
Luteolin	285	133	30	6.40
	285	151	20	
	285	175	20	
Quercetin	301	151	17	6.49
	301	179	11	
	301	121	25	
Pinoresinol	357	151	13	6.62
	357	136	27	
	357	92	39	
Apigenin	269	117	29	7.02
	269	151	20	
	269	149	19	
Tt-cinamic acid	147	103	7	7.08
	147	77	18	

## Appendix B

**Table A2:** Model-estimated marginal mean elemental concentrations for the seven studied *Salicornia* species. Estimated marginal means (EMMs) and 95% confidence intervals were obtained from models fitted to log_10_-transformed plant-level concentrations and were subsequently back-transformed to the original concentration scale (mg kg^−1^ dry weight). For all elements except As, models included species as a fixed effect and sampling site as a random intercept; As was analyzed using a linear model because the sampling-site variance was estimated as zero. Back-transformed EMMs represent model-based estimates on the original scale and should not be interpreted as arithmetic sample means.

Element	Species	Estimated Marginal Mean [95% CI]
Na	*S. alpini*	67,159 [36,055–125,094]
Na	*S. carinata*	67,335 [24,567–184,555]
Na	*S. fruticosa*	107,713 [38,706–299,745]
Na	*S. hispanica*	75,554 [27,724–205,899]
Na	*S. lagascae*	114,674 [41,208–319,117]
Na	*S. perennis*	134,315 [48,266–373,775]
Na	*S. pruinosa*	45,221 [26,537–77,059]
Mg	*S. alpini*	6517 [3210–13,231]
Mg	*S. carinata*	20,933 [5747–76,242]
Mg	*S. fruticosa*	11,942 [3183–44,802]
Mg	*S. hispanica*	16,477 [4443–61,109]
Mg	*S. lagascae*	16,714 [4455–62,705]
Mg	*S. perennis*	15,275 [4072–57,307]
Mg	*S. pruinosa*	4083 [2090–7979]
K	*S. alpini*	10,513 [4809–22,986]
K	*S. carinata*	17,906 [4259–75,280]
K	*S. fruticosa*	20,584 [4745–89,295]
K	*S. hispanica*	8491 [1981–36,392]
K	*S. lagascae*	31,930 [7360–138,513]
K	*S. perennis*	15,556 [3586–67,482]
K	*S. pruinosa*	4175 [1986–8775]
Ca	*S. alpini*	10,372 [6872–15,655]
Ca	*S. carinata*	7307 [3402–15,695]
Ca	*S. fruticosa*	13,453 [7361–24,584]
Ca	*S. hispanica*	14,669 [8399–25,620]
Ca	*S. lagascae*	18,401 [10,069–33,628]
Ca	*S. perennis*	15,918 [8711–29,090]
Ca	*S. pruinosa*	4497 [3266–6193]
Mn	*S. alpini*	34.0 [15.9–72.8]
Mn	*S. carinata*	35.6 [10.5–121]
Mn	*S. fruticosa*	28.8 [8.04–103]
Mn	*S. hispanica*	20.2 [5.81–70.1]
Mn	*S. lagascae*	23.1 [6.47–82.8]
Mn	*S. perennis*	93.9 [26.3–336]
Mn	*S. pruinosa*	32.9 [17.0–63.8]
Fe	*S. alpini*	571 [247–1320]
Fe	*S. carinata*	170 [31.2–933]
Fe	*S. fruticosa*	405 [115–1421]
Fe	*S. hispanica*	213 [63.1–722]
Fe	*S. lagascae*	543 [155–1906]
Fe	*S. perennis*	4894 [1393–17,188]
Fe	*S. pruinosa*	293 [150–573]
Ni	*S. alpini*	10.1 [3.78–26.8]
Ni	*S. carinata*	0.895 [0.187–4.28]
Ni	*S. fruticosa*	7.18 [1.40–37.0]
Ni	*S. hispanica*	2.78 [0.562–13.8]
Ni	*S. lagascae*	2.98 [0.579–15.3]
Ni	*S. perennis*	7.53 [1.46–38.7]
Ni	*S. pruinosa*	2.65 [1.13–6.20]
Cu	*S. alpini*	13.6 [4.83–38.3]
Cu	*S. carinata*	5.99 [1.20–29.9]
Cu	*S. fruticosa*	9.17 [1.65–51.0]
Cu	*S. hispanica*	14.3 [2.70–75.4]
Cu	*S. lagascae*	9.03 [1.62–50.3]
Cu	*S. perennis*	11.8 [2.11–65.4]
Cu	*S. pruinosa*	7.39 [3.03–18.0]
Zn	*S. alpini*	30.3 [15.1–60.6]
Zn	*S. carinata*	13.6 [4.87–38.3]
Zn	*S. fruticosa*	31.6 [10.7–93.4]
Zn	*S. hispanica*	29.5 [10.5–83.1]
Zn	*S. lagascae*	24.6 [8.31–72.6]
Zn	*S. perennis*	39.9 [13.5–118]
Zn	*S. pruinosa*	41.5 [23.4–73.3]
As	*S. alpini*	0.711 [0.131–3.87]
As	*S. carinata*	0.137 [0.041–0.453]
As	*S. fruticosa*	0.919 [0.084–10.1]
As	*S. hispanica*	0.713 [0.131–3.88]
As	*S. lagascae*	0.313 [0.029–3.44]
As	*S. perennis*	4.19 [0.381–46.0]
As	*S. pruinosa*	4.10 [1.24–13.6]
Sr	*S. alpini*	25.0 [3.65–171]
Sr	*S. carinata*	65.5 [2.42–1774]
Sr	*S. fruticosa*	65.7 [2.16–1994]
Sr	*S. hispanica*	200 [6.92–5767]
Sr	*S. lagascae*	579 [19.1–17,578]
Sr	*S. perennis*	415 [13.7–12,580]
Sr	*S. pruinosa*	13.1 [2.27–74.9]
Ba	*S. alpini*	10.9 [4.60–25.8]
Ba	*S. carinata*	2.02 [0.218–18.7]
Ba	*S. fruticosa*	5.13 [1.52–17.4]
Ba	*S. hispanica*	1.96 [0.793–4.83]
Ba	*S. lagascae*	7.52 [2.22–25.5]
Ba	*S. perennis*	59.8 [17.7–202]
Ba	*S. pruinosa*	5.08 [2.74–9.40]
Pb	*S. alpini*	1.54 [0.466–5.10]
Pb	*S. carinata*	0.250 [0.000–1100]
Pb	*S. fruticosa*	0.931 [0.167–5.19]
Pb	*S. hispanica*	0.910 [0.185–4.48]
Pb	*S. lagascae*	0.992 [0.178–5.52]
Pb	*S. perennis*	4.76 [0.855–26.5]
Pb	*S. pruinosa*	6.44 [2.59–16.0]
